# Sensing Mechanism and Excited-State Dynamics of a Widely Used Intracellular Fluorescent pH Probe: pHrodo

**DOI:** 10.1021/acs.jpclett.3c02653

**Published:** 2023-11-15

**Authors:** Simin Jiang, Yanmei He, Jonas Højberg Brandt, Li Zhao, Junsheng Chen

**Affiliations:** † Nano-Science Center & Department of Chemistry, University of Copenhagen, Universitetsparken 5, DK-2100 Copenhagen, Denmark; ‡ Division of Chemical Physics and NanoLund, Lund University, P.O. Box 124, 22100 Lund, Sweden; § College of Science, China University of Petroleum (East China), Qingdao 266580, Shandong, China

## Abstract

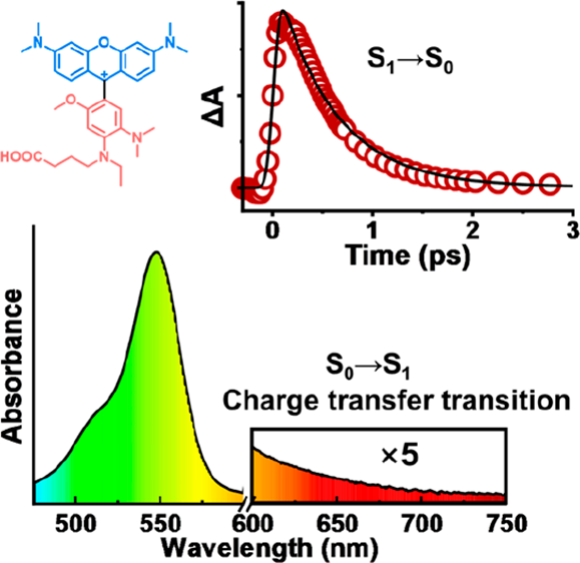

The pHrodo with an “off–on” response to the changes of pH has been widely used as a fluorescent pH probe for bioimaging. The fluorescence off–on mechanism is fundamentally important for its application and further development. Herein, the sensing mechanism, especially the relevant excited-state dynamics, of pHrodo is investigated by steady-state and time-resolved spectroscopy as well as quantum chemical calculations, showing that pHrodo is best understood using the bichromophore model. Its first excited state (S_1_) is a charge transfer state between two chromophores. From S_1_, pHrodo relaxes to its ground state (S_0_) via an ultrafast nonradiative process (∼0.5 ps), which causes its fluorescence to be “off”. After protonation, S_1_ becomes a localized excited state, which accounts for the fluorescence being turned “on”. Our work provides photophysical insight into the sensing mechanism of pHrodo and indicates the bichromophore model might be relevant to a wide range of fluorescent probes.

Fluorescent probes act as great analytical tools for real-time monitoring of biological parameters and processes such as temperature, pH, ion concentration, and reactive oxygen species, which are invisible or inaccessible to the human eye.^1−4^ Among them, the intracellular pH value, regulated by cell membrane proton pumps, is one of the most important to monitor because of its direct association with many pathological and physiological processes (e.g., enzyme activity, protein degradation, and mutations), the functions of organelles, and diseases (e.g., cancer, diabetes, and Alzheimer’s disease).^5−8^ Hence, it is important to be able to detect intracellular pH in real time and improve our understanding of how these pH-mediated changes affect migration and metastasis processes of different cells as well as how they lead to diseases.^9,10^

A large number of fluorescent pH probes have been developed on the basis of existing fluorophores, such as rhodamine, fluorescein, cyanine, triangulenium, bodipy, etc., to probe intracellular pH.^11−13^ Among the developed fluorescent pH probes, pHrodo with a rhodamine-like structure is a commercially available and widely used fluorescent pH probe for monitoring pH values in biological systems,^14,15^ such as monitoring the activity of the prototypic proton-pumping P-type adenosine triphosphatases at the single-molecule level,^16^ detection of acidic regions due to atherosclerotic lesions, and understanding how bitter taste receptors function in signaling for the immune system to activate airway diseases.^17^ Despite its wide applications, the sensing mechanism of pHrodo, especially its excited-state dynamics, is not yet well understood. A comprehensive understanding of its sensing mechanisms is beneficial for the further application and development of new fluorescent probes.^1,18−21^ As shown in Figure 1, the chemical structure of pHrodo (determined by Ogawa and co-workers on the basis of mass spectroscopic analysis and ^1^H and ^13^C NMR)^22^ is similar to that of aminorhodamine (ARh), which shows a fluorescence off–on switch with a change in pH^23−26^ and can function as a fluorescent pH probe, as well. For ARh, a detailed study revealed their sensing mechanism can be well understood by a bichromophore model.^23−25^ In the bichromophore model, the lowest excited state [the first excited state (S_1_)] is formed by strong coupling between two chromphores and is a weakly transition-allowed charge transfer (CT) state, which can be excited from the ground state (S_0_). This is different from the intramolecular charge transfer (TICT) model in which the TICT state is formed via structural reorganization typically from a localized state (LE); these are two local minima on the same potential energy surface.^27,28^ The weakly allowed S_1_ with CT character causes fluorescence quenching (off) of the ARh. Upon protonation (decrease in pH), their electronic structure is changed, and the lowest excited state is localized on the diamion–xanthenium chromophore, which ensures the fluorescence “turn on”. Considering the high degree of similarity in chemical structure between ARh and pHrodo, the presence of diamion–xanthium, and arylpyrylium parts in an analogous way, we would propose that the sensing mechanism of pHrodo might be understood as the bichromophore model, as well.

**Figure 1:**
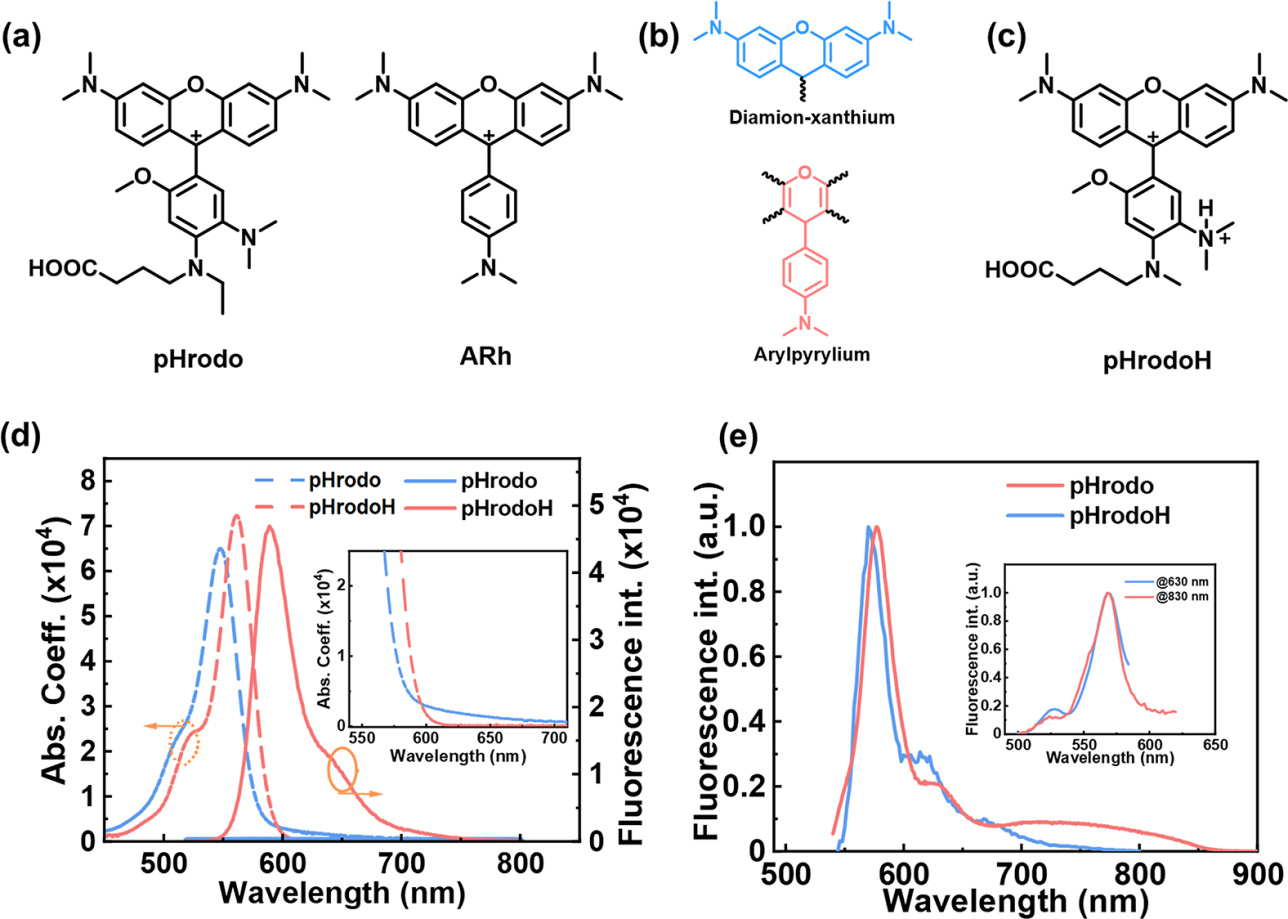
(a) Chemical structure of pHrodo and ARh. (b) Two chromophore units present in ARh: blue for the rhodamine-like diamino–xanthenium chromophore and red for the arylpyrylium chromophore. (c) Chemical structure of pHrodoH. (d) Absorption (dashed line) and fluorescence (solid line; λ_ex_ = 510 nm) spectra of pHrodo (blue) and pHrodoH [red, pHrodo protonated upon addition of 5 equiv of trifluoroacetic acid (TFA)] in acetonitrile, respectively. (e) Fluorescence of pHrodo and pHrodoH at 77 K. The inset shows the excitation spectra of pHrodo at 77 K, detected at 630 and 830 nm, respectively.

Herein, combining experimental with computational approaches, we investigate the sensing mechanism, specifically for the excited-state photophysical processes of pHrodo. Steady-state absorption spectroscopy was used to investigate the low-lying electronic transitions in which we observed a weak absorption tail in the long-wavelength region of the spectrum of pHrodo (Figure 1d). This indicates that there is a low-lying electronic transition with a low absorption coefficient, which can be attributed to a weakly allowed transition. Computational studies using density functional theory (DFT) and time-dependent DFT (TDDFT) were performed to complement the experimental results and support the existence of a weakly allowed low-lying electronic state, which agrees with the bichromophore model proposed in ARh.^23−25^ Femtosecond transient absorption (fs-TA) spectroscopy was used to study the relevant excited-state dynamics, which confirmed the existence of the low-lying excited state with a short lifetime (0.5 ps), accounting for the fluorescence quenching of pHrodo. Upon protonation, the electronic structure of pHrodo is changed, and the lowest excited state is localized on the diamino–xanthenium chromophore with a lifetime of ∼1.6 ns, which accounts for the fluorescence “turn on”. This study is the first exploration of the sensing mechanism and excited-state dynamics of pHrodo and beneficial for its further applications as well as developing new fluorescent probes based on the bichromophore model.

To focus on the sensing mechanism, excited-state dynamics and minimize the interaction between the solvent and pHrodo molecule, we performed all of the relevant optical spectroscopy measurements in acetonitrile (MeCN), which is free from hydrogen bond interaction compared to water. The main absorption band of pHrodo is located at the visible region with a peak centered at 548 nm (Figure 1d). A weak absorption tail in the range of 600–700 nm is present (Figure 1d, inset), which is also observed in ARh and has been assigned to the weakly allowed S_0_ → S_1_ transition with CT character.^23−25^ Under 510 nm excitation, no clear fluorescence was observed in pHrodo. Upon addition of adequate trifluoroacetic acid (TFA) in pHrodo, the aminophenyl group of pHrodo can be protonated. Protonation can happen at the *meta* and *para* positions, but we do not try to distinguish which one of them is preferred here, as they show a similar electronic structural change based on DFT/TDDFT calculations (see the sections below). For the sake of illustration, we assign the amine at the *meta* position to be protonated and name it pHrodoH (Figure 1c). The main absorption band of pHrodoH is red-shifted (peak at 561 nm) compared to that of pHrodo [the absorption coefficient (ε) has been calculated on the basis of the maximum ε of 65 000 M^–1^ cm^–1^ from ref (29)]. Furthermore, the weak absorption tail in the range of 600–700 nm disappeared in pHrodoH. Under 510 nm excitation, pHrodoH shows a strong fluorescence with a fluorescence quantum yield (FQY) of 40.1 ± 0.1%.

As pHrodo is not fluorescent at room temperature (Figure 1d), it is almost impossible to access the electronic structure of its excited state based on steady-state fluorescence and excitation spectra. At low temperatures (e.g., 77 K, cryogenic temperature), the nonradiative decay pathways can be effectively inhibited and the fluorescence emission process becomes possible. This has been observed in ARh and its derivatives,^23,24^ in which the change of the dihedral angle between xanthenium and phenyl groups is limited and ARh becomes fluorescent at 77 K (frozen condition). Motivated by the fluorescence “turn on” at low temperatures, we measured the fluorescence emission of pHrodo and pHrodoH at 77 K in 2-methyltetrahydrofuran (2-MeTHF). Here we used 2-MeTHF, because it can form transparent glass for low-temperature measurements. As shown in Figure 1e, pHrodo shows a main emission band in the range of 550–650 nm similar to that of pHrodoH, accompanied by a weak and broad shoulder emission in the longer-wavelength region of 700–850 nm. Meanwhile, the nearly identical excitation spectra detected at 630 and 830 nm suggest that the two emission bands originate from the same species. Considering the analogical structure of pHrodo and ARh, we could make an analogous assignment that the main emission in the range of 550–650 nm might come from the S_2_ → S_0_ transition and the shoulder emission might be the S_1_ → S_0_ transition.^23,24^

To gain insight into the nature of the low-lying excited states, we then explored the electronic structure and the relevant electronic transitions of pHrodo and pHrodoH by DFT and TDDFT quantum chemical calculations. As shown in Figure S2, the highest occupied molecular orbital (HOMO) of pHrodo is located on the arylpyrylium chromophore part, while the lowest unoccupied molecular orbital (LUMO) is spread on the diamion–xanthium chromophore part. The nearly separated HOMO and LUMO leads to the obvious CT character of S_1_ in space with a relatively low oscillator strength (Table S1). In addition, the dihedral angle of pHrodo between diamion–xanthium and arylpyrylium changes significantly from 62.9° for S_0_ to 107.7° for S_1_. According to the optimized geometry of S_0_ and S_1_, we further calculated the large root-mean-square deviation (RMSD) (details in the Supporting Information) between the two states. The relatively large RMSD of 1.41 Å indicates the considerable excited-state structural change, which can contribute to the nonradiative decay process of the excited state of pHrodo.^27,30,31^ Furthermore, the CT character of S_1_ is further confirmed by the calculated dipole moments of pHrodo (Table S2). pHrodo has a dipole moment (8.68 D) in S_0_ due to its cationic nature with charge (positive) localization on the diamion–xanthium part. In S_1_, the dipole moment of pHrodo is decreased to 6.87 D. The decrease in the dipole moment (from S_0_ to S_1_) is due to the charge (negative) transfer from the arylpyrylium chromophore part to the cationic diamion–xanthium part in S_1_. Upon protonation (formation of pHrodoH), its electronic structure is changed. The amine group at the *meta* or *para* position can be protonated. In our calculation, we considered both scenarios and found the energy of *meta* position protonation is lower than that of *para* position protonation (details in the Supporting Information). The energy difference is 5.64 kcal/mol, which indicates that the *meta* position is preferred. Considering we have added excess TFA during the experiment, we would expect both conformations can exist in our experiment. By analyzing their low-lying electronic transitions, we found their electronic structures are extremely similar (Figures S4–S6 and Table S3). Thereafter, we use the *meta* position-protonated structure to represent pHrodoH. In pHrodoH, the lone electron pair of the nitrogen atom of the amine group attracts a proton and forms a stable covalent bond, which prevents the lone electron pair from participating in the molecular conjugation. This weakens the electron-donating ability of the amine group and then deepens the energy level of the arylpyrylium chromophore. Thus, for pHrodoH, the HOMO distribution is altered from arylpyrylium to the diamion–xanthium chromophore. The HOMO and LUMO are both spread on the diamion–xanthium part. This distribution of frontier molecular orbitals (FMOs), on one hand, increases the overlap of the HOMO and LUMO to enlarge the oscillator strength; on the other hand, the distribution reduces the degree of structural reorganization during the excited relaxation process. The calculated RMSD between S_1_ and S_0_ of pHrodoH is only 0.46 Å, which is only one-third of the calculated RMSD of pHrodo (Table S1). Meanwhile, due to the distribution of small FMOs on arylpyrylium, the free rotation of arylpyrylium plays a smaller role in molecular emission and cannot lead to a fast nonradiation process like that in pHrodo.^32^ Combined with the enlarged oscillator strength, the significant improvement in FQY for pHrodoH could be expected, which agrees with the measured FQY of pHrodoH being ∼40%. Noticeably, the S_2_ → S_0_ transition of pHrodo from LUMO to HOMO–1 is similar to the S_1_ → S_0_ transition of pHrodoH, which provides the theoretical prediction for the emission origination of pHrodo in the region of 550–650 nm at 77 K (Figure 1e).

The steady-state spectra and DFT/TDDFT calculations are informative, but to understand the quenched fluorescence in pHrodo, it is necessary to conduct time-resolved experiments to reveal photoinduced excited-state dynamics. Fluorescence quenching typically involves ultrafast processes. To gain a deep understanding of these processes, spectroscopic techniques with sufficient resolution are needed to resolve the dynamics taking place as excited pHrodo relaxes from highly excited state (S_*n*_, where *n* ≥ 2) to S_1_, and the S_1_ goes back to S_0_. Our analysis relies on optical “pump–probe” methods, specifically fs-TA spectroscopy, enabling us to track spectral changes with time resolution as fine as hundreds of femtoseconds.

The fs-TA spectra of pHrodo shown in panels a and b of Figure 2 were recorded by pumping pHrodo with a sub-100 fs pulsed laser peak centered at 525 nm and probing with supercontinuum white light. The fs-TA spectra of pHrodo share similar features with the reported fs-TA spectra of TMARh.^25^ A broad negative peak centered at approximately 550 nm corresponds to the signal associated with the ground-state bleach (GSB) (Figure 2a,b), which aligns well with the steady-state absorption spectrum (Figure 1d). There seems to be a weak stimulated emission (SE) signal between 570 and 650 nm, which matches the low-temperature fluorescence spectrum of pHrodo (Figure 1e). As shown in Figure 2c, the SE signal trace at 625 nm recovers to zero within 1 ps, which is much faster than the recovery of the GSB signal at 555 nm. This SE signal might have originated from the S_2_ to S_0_ transition, a band distinctly originating from the diamino-xanthium. Following photoexcitation, we observed an excited-state absorption (ESA) signal with a peak emerging at ∼450 nm. Over time, the ESA signal at 450 nm decreases, while a new ESA signal with a peak around 440 nm emerges. The spectral evolution indicates a fast relaxation process has happened on the time scale of the instrument response function (IRF ∼ 100 fs). However, we did not observe a clear isosbestic point due to the fast spectral evolution. A similar spectral evolution has been observed in TMARh and has been assigned to internal conversion from S_2_ to S_1_.^25^ However, it is difficult to make a similar unambiguous assignment considering the ∼100 fs IRF. These signals are readily evident in the kinetic traces shown in Figure 2c and Figure S7. Immediately following photoexcitation, we observed the ESA signal at 480 nm followed by a fast decay, while the ESA signal at 440 nm undergoes a building-up process (Figure S7) over the first 100 fs followed by a relatively slower decay compared to the ESA signal at 480 nm. All of the signals mentioned above disappear within ∼3 ps.

**Figure 2:**
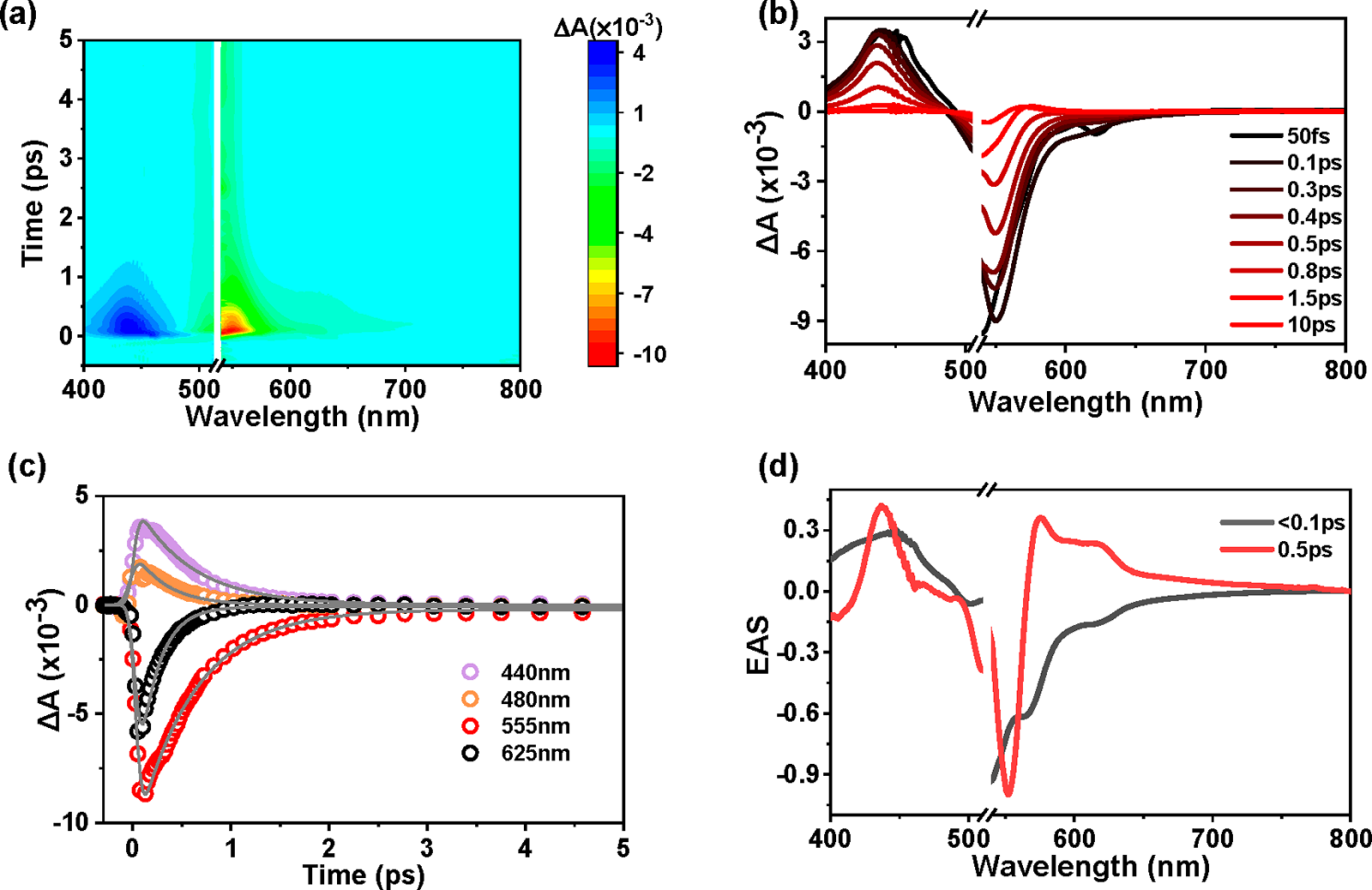
(a) Pseudocolor representation of the fs-TA spectra of pHrodo under 525 nm excitation. (b) fs-TA spectra of pHrodo as a function of time delay. (c) Kinetic traces at 440, 480, 555, and 625 nm, in which the gray curves are fitting results based on global analysis. (d) Normalized EAS spectra obtained from global analysis.

The qualitative analysis of the fs-TA spectra offers valuable insight into the excited-state dynamics of pHrodo, suggesting the potential involvement of two excited states following excitation with a 525 nm laser. For a more quantitative understanding of the dynamic process, we employed single-value decomposition (SVD) analysis to determine the number of excited states using the fs-TA data. SVD analysis indicates that it can be effectively characterized by two components, corresponding to two excited states (S_2_ and S_1_), which are formed after 525 nm photoexcitation. The assignment of two excited states matches well with the steady-state absorption spectrum (Figure 1d) and the TDDFT calculations (Table S1). Hence, following excitation with a 525 nm laser, a sequential model (S_2_ → S_1_ → S_0_) provides an effective description of the relaxation process, as the model imposes a step-by-step relaxation process, initially only one state is populated, and the population relaxes sequentially through all states. The sequential relaxation is further supported by the spectral evolution mentioned above (Figure 2a,b). The sum-of-exponential model with global time constants was employed for global analysis (details in the Supporting Information). The fitted kinetic curves match well with the fs-TA data (Figure 2c), which indicates the reliability of the global fit approach. The global analysis allows us to derive the lifetimes of S_2_ and S_1_ of the photoexcited pHrodo to be <100 fs and 0.5 ps, respectively. Figure 2d shows the obtained evolution-associated spectra (EAS) representing the spectra of S_2_ and S_1_. The ultrafast relaxation from the gray EAS to the red EAS is accompanied by replacement of the broad ESA peak at 450 nm with a relatively narrower ESA peak at 440 nm, which can be assigned to the S_2_ → S_1_ IC process. The red EAS represents the S_1_ of pHrodo with a lifetime of 0.5 ps.

**Figure 3:**
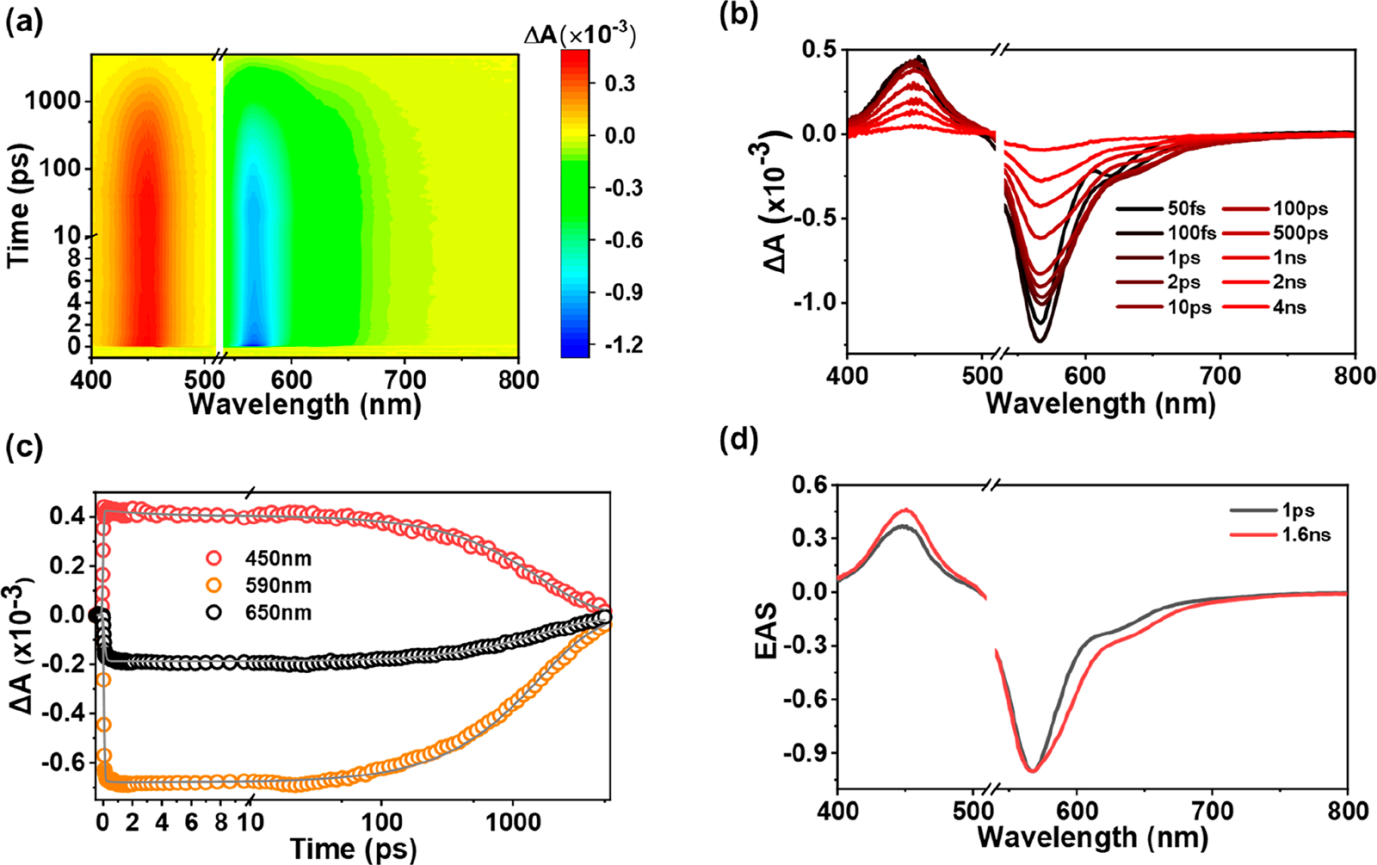
(a) Pseudocolor fs-TA data map of pHrodoH with excitation of 525 nm. (b) fs-TA spectra of pHrodoH as a function of time delay. (c) Kinetic traces at 450, 590, and 650 nm. The gray curves are from global fitting. (d) Normalized EAS spectra obtained from global fitting.

The existence of the weakly allowed S_0_ → S_1_ transition can be further confirmed by conducting the fs-TA experiment using an excitation pulse with a wavelength centered at 640 nm (Figure S8). The 640 nm laser pulse energy is only marginally higher than the onset of absorption spectra (Figure 1d); hence, the S_0_ → S_1_ transition can be directly accessed without the involvement of S_2_. In this scenario, we do not observe the ESA signal (from the S_2_ → S*_n_* transition) peak at 450 nm (Figure 2b). Instead, we can see the appearance of the ESA peak at 440 nm followed by 640 nm laser excitation. In fs-TA, the use of 640 nm light for directly exciting S_1_ provides additional evidence supporting the presence of a low-lying state (S_1_) characterized by a weakly allowed optical transition. Importantly, we can use a straightforward monoexponential analysis of decay across multiple wavelengths, resulting in the lifetime of S_1_ being 0.5 ps. This determination aligns remarkably well with the comprehensive analysis of the more intricate decay observed upon excitation at 525 nm. Considering pHrodo is nonfluorescent at room temperature, the process from S_1_ to S_0_ with such a short lifetime could be identified as the nonradiative recombination. The fast nonradiative recombination could be associated with the excited-state structural reorganization (Figure S3 and Table S1), charge redistribution, and the small energy gap (<1.5 eV) between S_1_ and S_0_ [according to the second fluorescence peak that extends over 800 nm (Figure 1e)]. The energy gap can be even smaller at room temperature, in which the structural relaxation is much less restricted than that at 77 K. On the basis of energy gap law, where the nonradiative rate exponentially increases with a decrease in the energy gap,^33,34^ the S_1_ of pHrodo can more readily return to S_0_ through the nonradiativere combination path. We noted that the nonradiative process of pHrodo (0.5 ps) is much faster than that of TMARh (1.7 ps),^25^ which might be related to the different molecular rigidity. Compared with TMARh (with four large steric hindrance methoxy groups^25^), only one methoxy group is present in pHrodo (Figure 1a). The decreased steric hindrance allows the carbon–carbon single bond between the xanthenium and phenyl groups to rotate more easily in pHrodo than in TMARh, which leads to molecular structural relaxation and a fast nonradiative decay process.

To further understand the pH sensing mechanism of pHrodo, we carried out a fs-TA experiment with pHrodoH (protonated form of pHrodo) under the same excitation condition (525 nm laser pulse). As shown in panels a and b of Figure 3, the GSB signal was centered at ∼560 nm, which matches with the steady-state absorption spectrum (Figure 1d) of pHrodoH. As expected, a strong stimulated emission (SE) signal is present between 570 and 650 nm, because of its strong fluorescence emission with a FQY of ∼40%. A positive ESA signal with a peak located at ∼450 nm is present right after the pump pulse excitation. We do not see any clear spectral evolution of the ESA signal, other than the decrease in the ESA signal intensity together with the GSB and SE (Figure 3b). As shown in Figure 3c, the fs-TA signals of pHrodoH decay several orders of magnitude slower than those of pHrodo (Figure 2). To determine the decay time constants, we carried out global fitting of the data set and obtained two time constants (1 ps and 1.6 ns) with their representative EAS shown in Figure 3d. The time constant of 1.6 ns can be ascribed to the final emissive state of pHrodoH, as it matches well with the fluorescence lifetime (1.8 ns) measured on the basis of time-correlated single-photon counting (Figure S9). The 1 ps time constant component has an EAS very similar to that of the 1.6 ns time constant, which indicates they most likely originated from the same electronic state. Considering the high FQY of pHrodoH, we could exclude the nonradiative process that has a 1 ps time constant. Hence, we can assign such a short lifetime component to a higher vibrational state as the excitation energy (525 nm, 2.36 eV) is much higher than the fluorescence photon energy (600 nm, 2.06 eV).

On the basis of the results presented above, the intrinsic excited-state dynamics of pHrodo and pHrodoH are illustrated in Figure 4. Upon 525 nm excitation, the high-energy S_2_ state will decay to S_1_ via a fast IC process (<0.1 ps). The excited pHrodo relaxes from S_1_ to S_0_ via a nonradiative process with a time constant of 0.5 ps, which involves both charge redistribution and structural reorganization. As a result, pHrodo shows fluorescence off. After protonation, the low-lying CT S_1_ will change to the LE state. Under 525 nm excitation, the higher-energy vibrational S_1_ state relaxes to the lowest S_1_ state in 1 ps. Because of the LE character of S_1_ with a large oscillator strength, excited pHrodoH decays to S_0_ within 1.6 ns to emit bright fluorescence with a fluorescence quantum yield of ∼40%.

**Figure 4:**
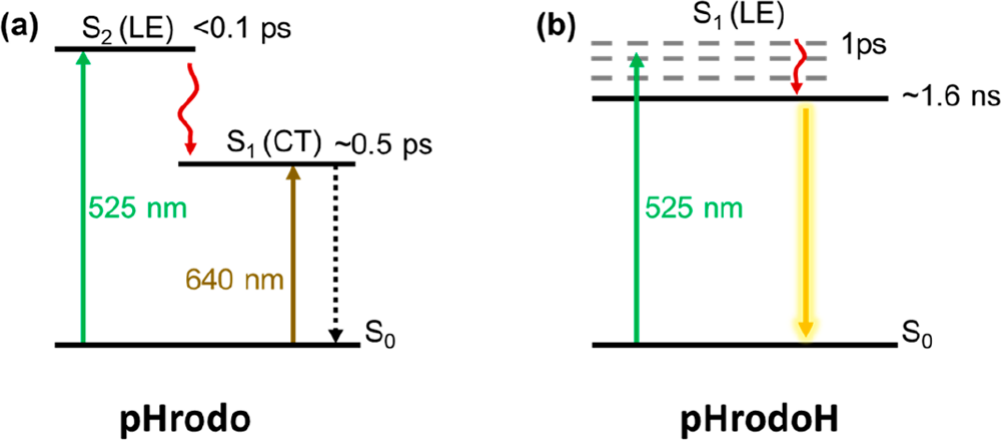
Schematic illustration of the excited-state dynamics in (a) pHrodo and (b) pHrodoH.

In summary, we investigated the sensing mechanism of pHrodo as a fluorescent “off–on” pH probe based on optical spectroscopy experiments and quantum chemical calculations. The steady-state absorption spectrum of pHrodo shows a weak absorption band in the range of 600–700 nm, which originates from a weakly allowed CT transition involving diamion–xanthium and arylpyrylium chromophores. Such an assignment is based on TDDFT calculations, which also reveal that the strongest absorption peak at 548 nm is the S_0_ → S_2_ transition localized on the diamion–xanthium chromophore. Hence, the pHrodo can be well understood by a bichromophore model, in which S_1_ is a CT state and accounts for the fluorescence “off” nature of pHrodo. The fs-TA measurement elucidates the fast IC process from S_2_ to S_1_ with a time constant of <100 fs, followed by nonradiative S_1_ to S_0_ relaxation with a time constant of 0.5 ps. Furthermore, the weakly allowed (S_0_ → S_1_) transition has been accessed in fs-TA measurements with a 640 nm laser and shows a fast decay process of S_1_ with a lifetime of 0.5 ps. Protonation causes a change in the electronic structure. The S_0_ → S_1_ transition with a large transition oscillator strength becomes localized on the diamion–xanthium chromophore, which ensures the fluorescence turn on. This study offers detailed insight into the sensing mechanism of the widely used fluorescent pH probe, pHrodo, examining it on both the ultrafast time scale and the molecular microscopic level. We anticipate that the findings regarding the photoinduced deactivation process presented in this research will be valuable for the application of pHrodo and future development of molecular fluorescent probes based on bichromophores.

## Supporting Information Available

Experimental details about materials and solvents, theoretical calculation method, technical details of ultraviolet–visible, PL, and TA spectroscopy, theoretical calculation results, fluorescence lifetime measurement of pHrodoH in MeCN, and TA spectra and kinetic traces of pHrodo with 640 nm excitation (PDF)
Transparent Peer Review report available (PDF)
