# Supplementary material for: Sensing Mechanism and Excited-State Dynamics of a Widely Used Intracellular Fluorescent pH Probe: pHrodo
Source: J Phys Chem Lett. 2023 Nov 15;14(46):10482–8. doi: 10.1021/acs.jpclett.3c02653 (PMC10683063; doi:10.1021/acs.jpclett.3c02653)
Supplement: Supplementary file 1 — jz3c02653_si_001.pdf [file jz3c02653_si_001.pdf]

Supporting Information

Sensing Mechanism and Excited-State Dynamics of a Widely Used Intracelluar Fluorescent pH Probe: pHrodo

Simin Jiang,[1] Yanmei He, [1, 2] Jonas Højberg Brandt,[1] Li Zhao,[3, *] Junsheng Chen[1, 2, *]

[1]*Nano-Science Center & Department of Chemistry, University of Copenhagen, Universitetsparken 5, DK-2100 Copenhagen, Denmark*
[2] *Division of Chemical Physics and NanoLund, Lund University, P.O. Box 124, 22100 Lund, Sweden*
[3] *College of Science, China University of Petroleum (East China), Qingdao 266580, Shandong, China*

* Corresponding authors
Li Zhao: zhaoli282@upc.edu.cn
Junsheng Chen: Junsheng.chen@chemphys.lu.se

Materials and Solvents

pHrodo™ was purchased from ThermoFisher Scientific. Trifluoroacetic acid (TFA, 99.9%) was purchased from abcr GmbH. Acetonitrile (HPLC) was purchased from VWR Chemical BDH. 2-Methyltetrahydrofuran (2-MeTHF, ≥ 99.0%) was purchased from Sigma-Alorich. All the materials and solvents were used directly without any further purification.

Theoretical calculation method

All of the simulations were performed using the Gaussian 09 program package.[1] Ground state (S_0) geometries were initially optimized using density functional theory (DFT) with the Cam-B3LYP functional[2] and 6-31g(d) basis set[3] in acetonitrile with polarizable continuum model (PCM) using integral equation formalism variant (IEF-PCM)[4, 5] . All the excited states were optimized based on S_0 geometries using time-dependent density functional theory (TDDFT) under Cam-B3LYP/6-31g(d) theoretical level in acetonitrile with PCM.

Root-mean-squared deviation (RMSD) calculation

RMSD value is a typical parameter to characterize the discrepancy between two structures, which can be expressed by the following formula:

$$\mathrm{RMSD} = \sqrt{\frac{\sum_{i}[(x_i - x_i^{'})^2 + (y_i - y_i^{'})^2 + [(z_i - z_i^{'})^2]}{N}} \qquad \text{(S1)}$$

Where x_i, y_i, z_i,and $x_i^{'}$, $y_i^{'}$, $z_i^{'}$, are the x, y, z axis coordinate of ith atom in the first and second structure, respectively, and N is the total number of atoms in the structure.

Dihedral angle calculation

Dihedral angle is defined by the planes between arylpyrylium and diamion-xanthium. In our analysis, we drew two planes, which cross arylpyrylium and diamion-xanthium, and we measured the angle between the two planes.

Photo-physical characterization

Steady state absorption spectra were measured by Cary 5000 UV/Vis/NIR spectrophotometer. Steady state fluorescence spectra and fluorescence decay were measured by FluoTime 300. Fluorescence quantum yield (FQY) was calculated at the excitation wavelength of 510 nm, with respect to Rhodamine 6G (FQY=0.95) as the reference.[6]

$$FQY_s = PLQY_{\mathrm{r}}\left(\frac{m_s}{m_r}\right)\left(\frac{n_s}{n_r}\right)^2$$

Where *m* is the slop of the plot of integrated fluorescence intensity against absorbance (Figure S1 c and f); *n* is the refractive index of solvent, ethanol (n_s=1.363) for Rhodamine 6G and acetonitrile (n_r=1.325) for pHrodoH. The subscripts '*r*' and '*s*' refer to Rhodamine 6G and pHrodoH, respectively.

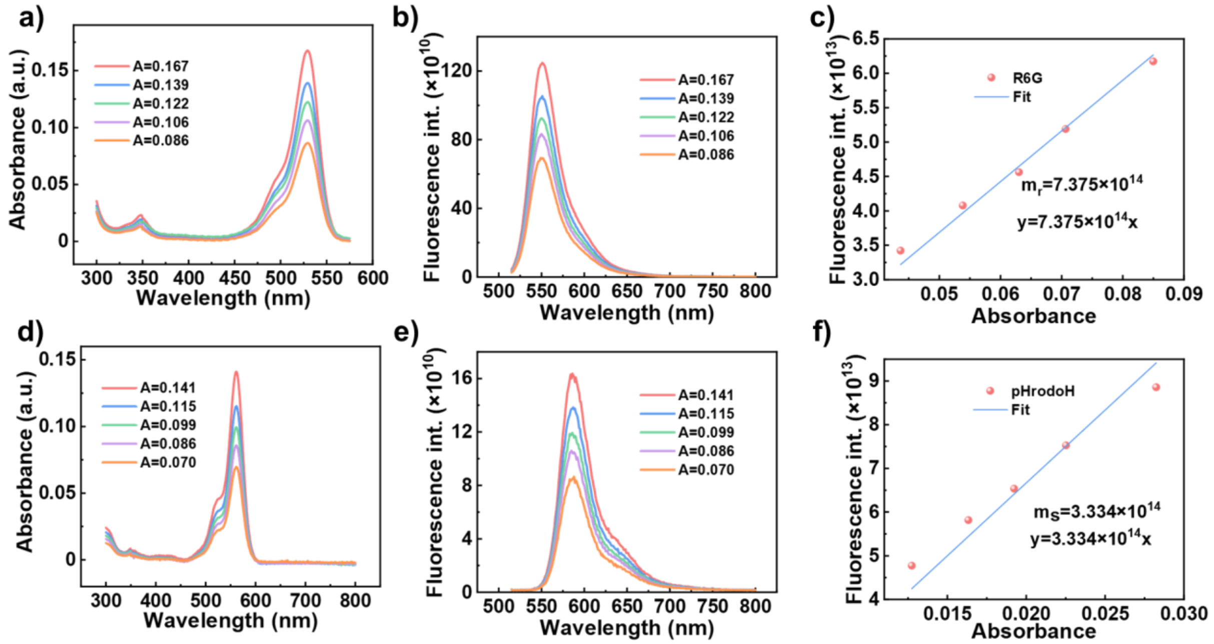

Figure S1. (a-c) UV-vis absorption, fluorescence spectra and the fluorescence intensity-absorbance curve of Rhodamine 6G; (d-f) UV-vis absorption, fluorescence spectra and the fluorescence intensity-absorbance curve of pHrodoH.

Femtosecond transient absorption (fs-TA) spectroscopy

fs-TA experiments were performed by using a femtosecond pump–probe setup. Laser pulses (796 nm, 60 fs pulse length, 4 kHz repetition rate) were generated using a regenerative amplifier (Solstice Ace) seeded by a femtosecond oscillator (Mai Tai SP, both Spectra Physics). For the pump, we used the TOPAS C (Light Conversion) to obtain pulses with a central wavelength located at 525 nm or 640 nm. The pump pulse energies were set to 0.3 μJ and 2.0 μJ per pulse for 525 nm and 640 nm, respectively. The spot size was approximately 0.2 mm^2 for both pump wavelengths. For the probe, we used supercontinuum generation from a thin CaF_2 plate. The mutual polarization between the pump and probe beams was set to the magic angle (54.7°) by placing a Berek compensator in the pump beam. There is no photodegradation after fs-TA experiments by checking the steady-state absorption spectra. The global analysis was performed by Glotaran software package (http://glotaran.org). The simple sequential decay model with two components was used to do SVD global fitting.[7]

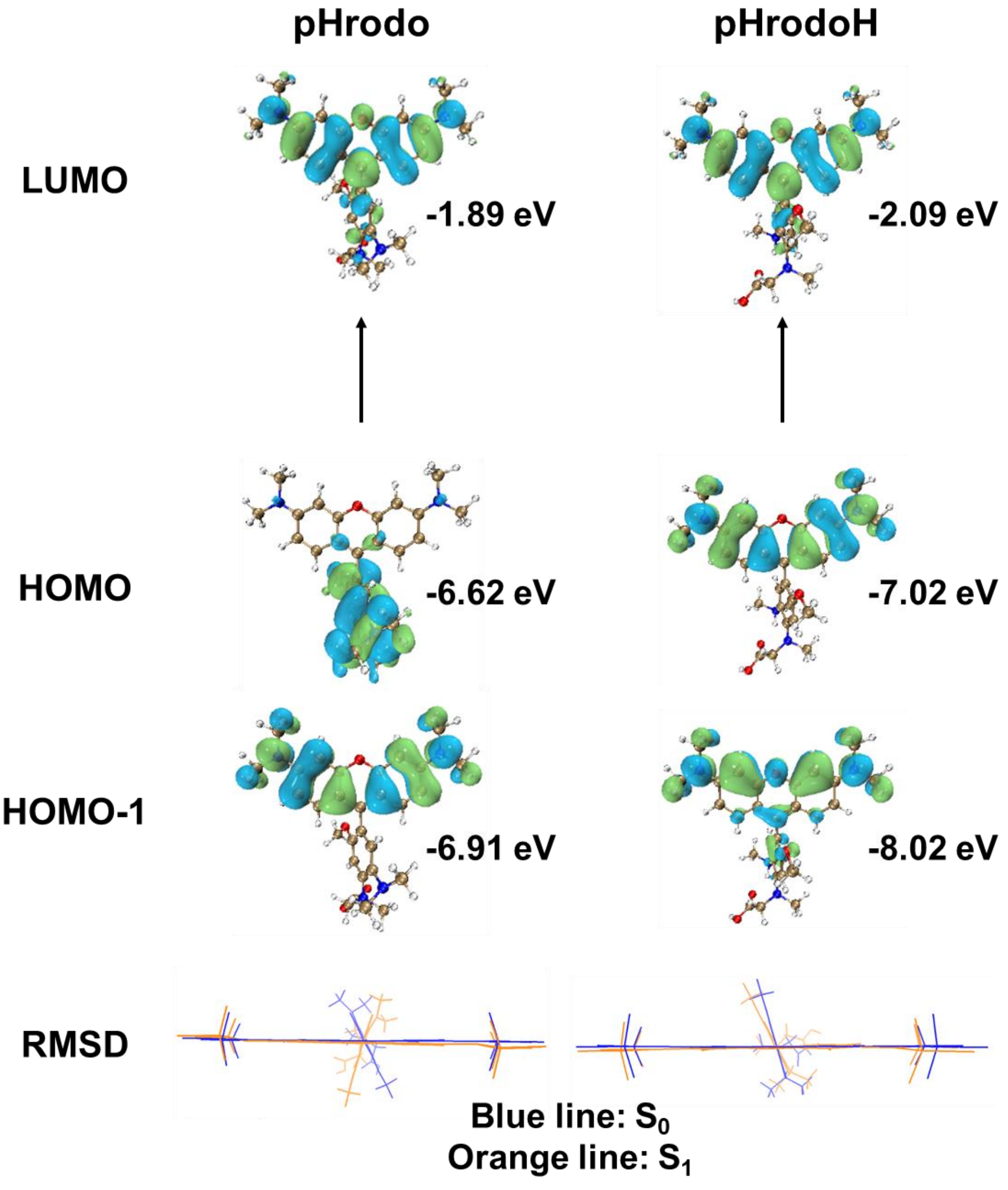

Figure S2. Computational frontier molecular orbitals (HOMO: highest occupied molecular orbital, LUMO: lowest unoccupied molecular orbital) and RMSD between S_0 (in blue color) and S_1 (in orange color) for pHrodo and pHrodoH.

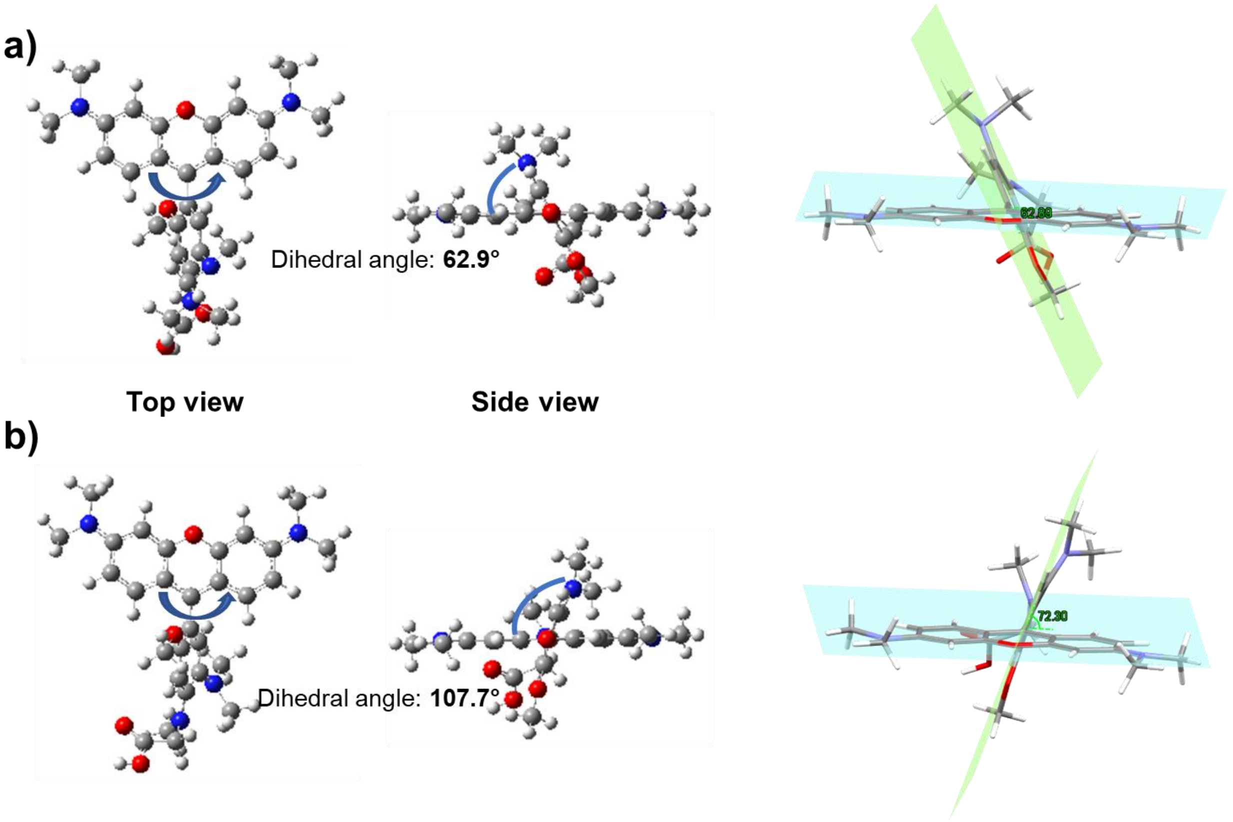

Figure S3. The optimized geometries and dihedral angle of pHrodo for (a) S_0 state and (b) S_1 state.

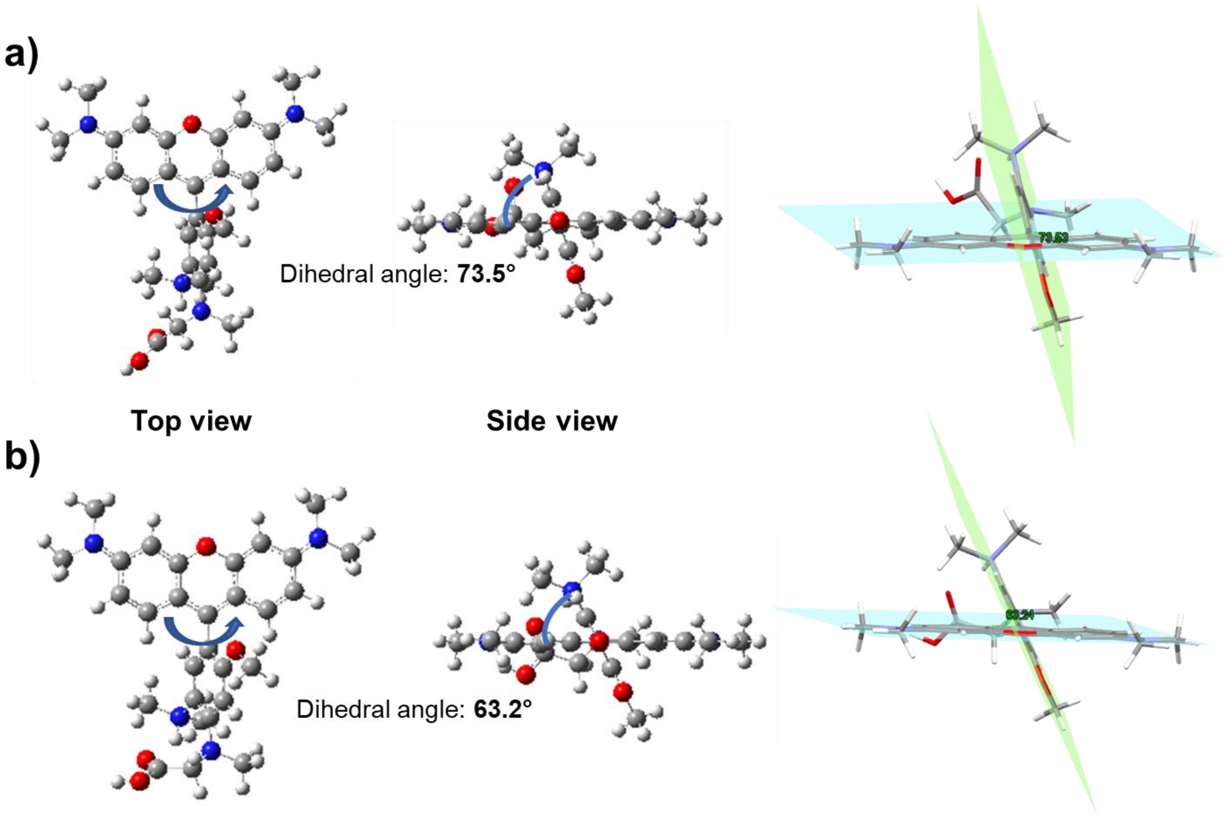

Figure S4. The optimized geometries and dihedral angle of pHrodoH protonated at *meta*-position for (a) S_0 state and (b) S_1 state.

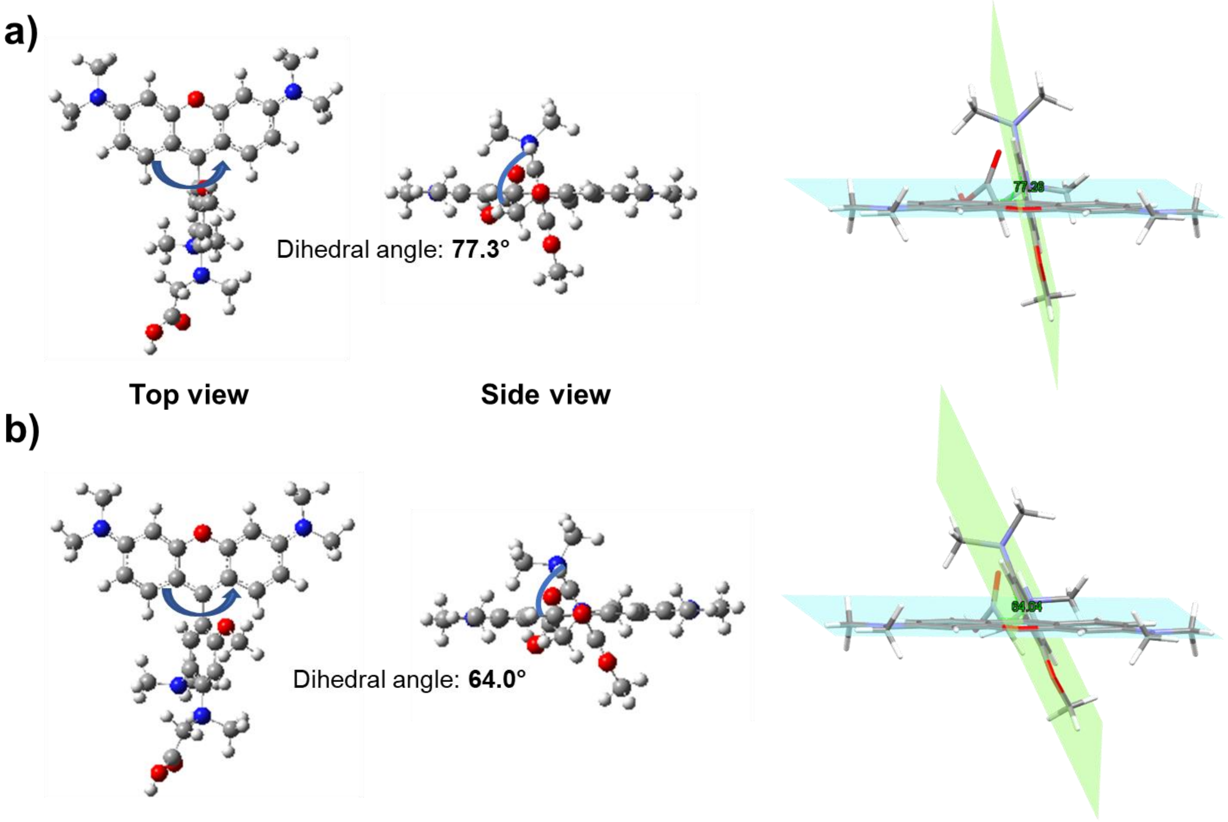

Figure S5. The optimized geometries and dihedral angle of pHrodoH protonated at *para*-position for (a) S_0 state and (b) S_1 state.

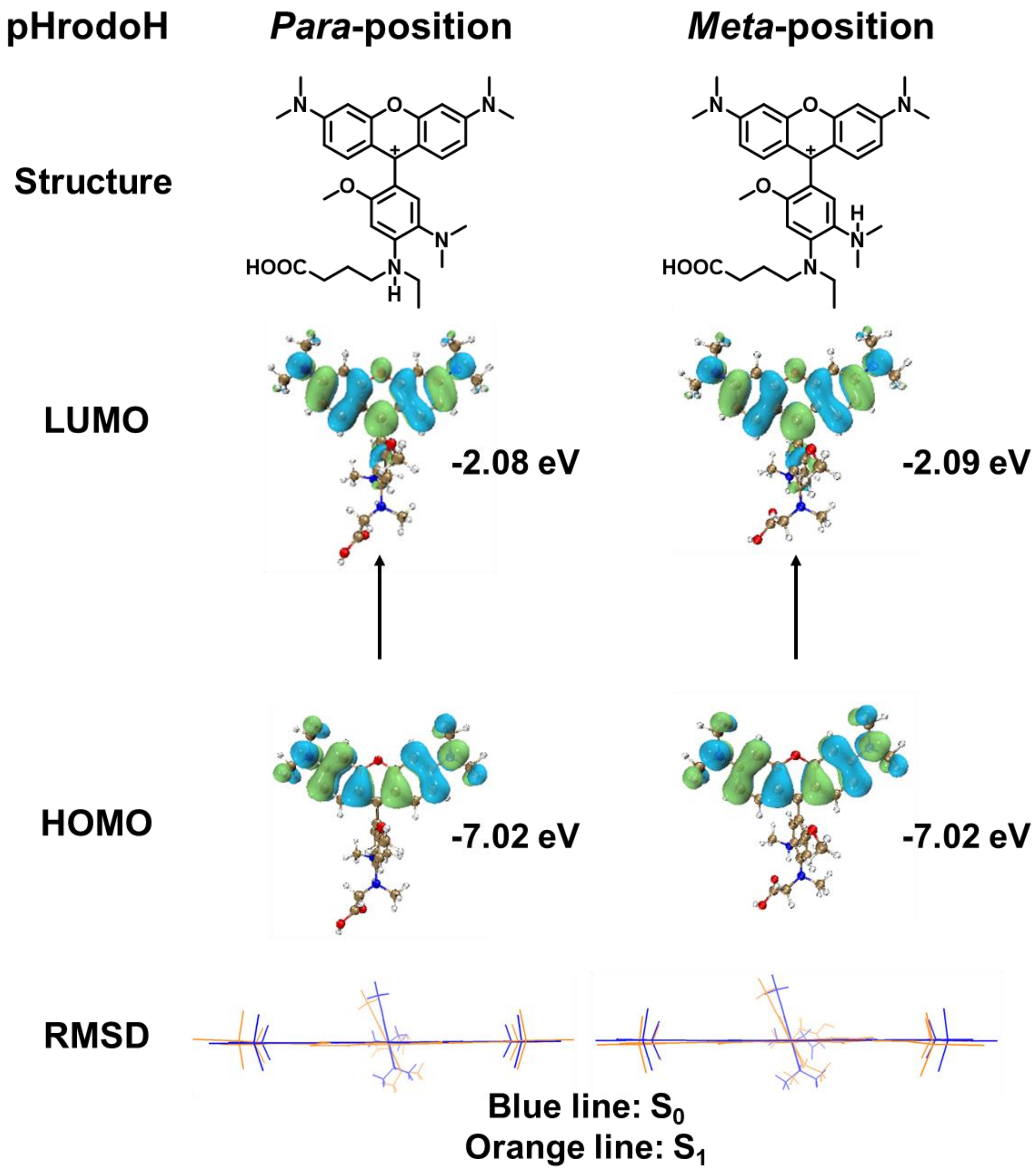

Figure S6. Computational frontier molecular orbitals and RMSD between S_0 (in bule color) and S_1 (in orange color) for pHrodoH with different protonated positions.

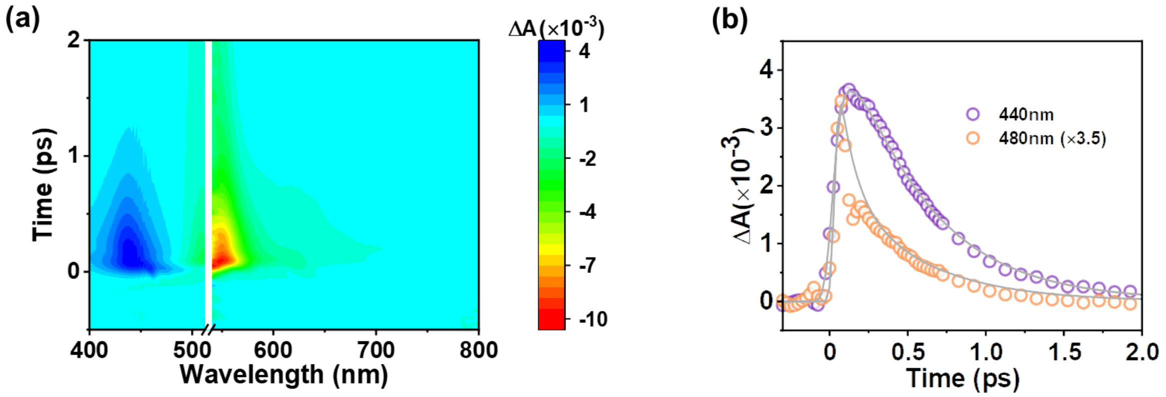

Figure S7. (a) Pseudocolor representation of the fs-TA spectra of pHrodo under the excitation of 525 nm zoomed within 2 ps. (b) The kinetics traces at 440 and 480nm zoomed within 2 ps.

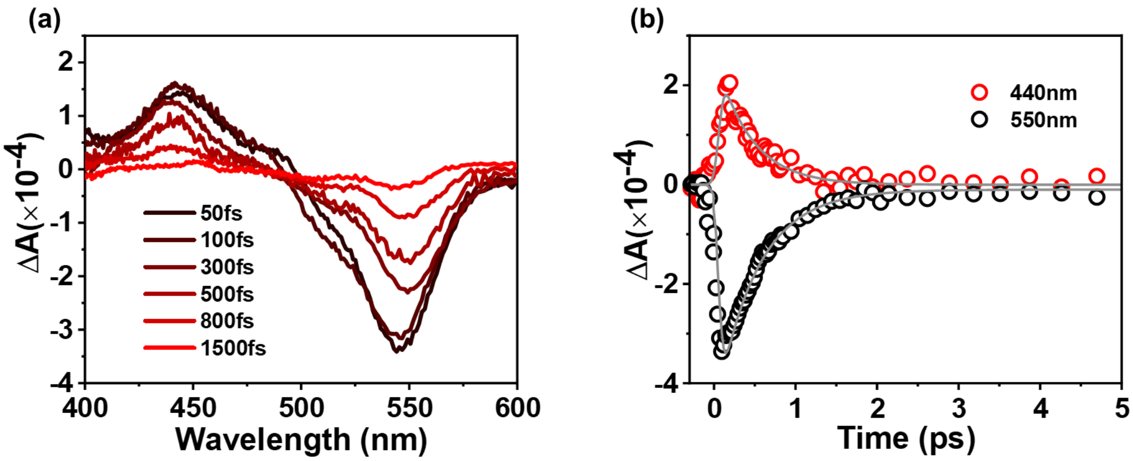

Figure S8. (a) The fs-TA spectra of pHrodo™ as a function of time delay with 640 nm excitation. (b) The kinetics traces at 440 and 550 nm, respectively.

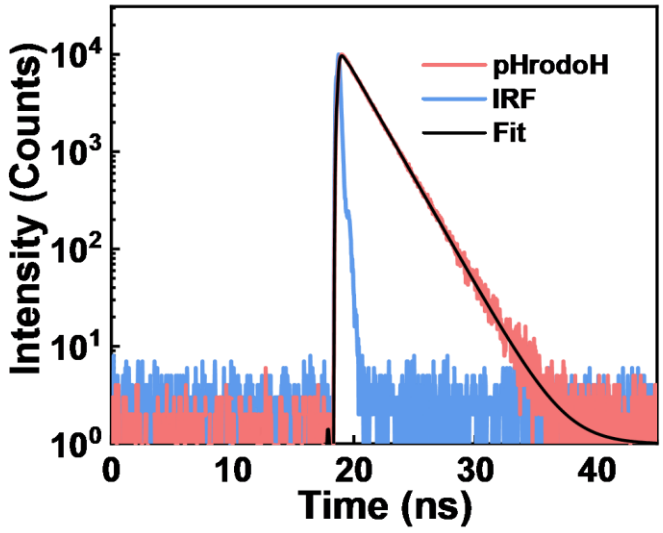

Figure S9. Fluorescence lifetime of pHrodoH measured in MeCN.

Table S1. Summary of calculated data for pHrodo and pHrodoH.

Compounds	Electronic transition	Energy (nm/eV)	Oscillator strength	Contribution	RMSD (Å)
**pHrodo**	$S_0 \rightarrow S_1$	436/2.68	0.16	H→L	1.41
	$S_0 \rightarrow S_2$	427/2.90	1.02	H-1→L	-
**pHrodoH**	$S_0 \rightarrow S_1$	437/2.84	1.06	H→L	0.46

Table S2. Dipole moment of pHrodo and pHrodoH at S_0 and S_1.

Compounds	Dipole moment of S_0 (D)	Dipole moment of S_1 (D)
**pHrodo**	8.68	6.87
**pHrodoH**	5.85	6.65

Table S3. Summary of calculated data for pHrodoH with different protonated positions.

Position	S_0 Energy (Hartree)	Electronic transition	Energy (nm/eV)	Oscillator strength	Contribution	RMSD[a] (Å)
Para	-1645.508	$S_0 \rightarrow S_1$	435/2.85	1.07	H→L	0.50
Meta	-1645.517	$S_0 \rightarrow S_1$	437/2.84	1.06	H→L	0.46

[a] Root Mean Square Deviation (RMSD) between the optimized structure of S_0 and S_1.

Table S4. Optimized geometry based on DFT calculation for **pHrodo** in S_0 state.

C	-3.2699	3.52424	-0.52068
C	-3.86787	2.25629	-0.37571
C	-3.07864	1.14183	-0.20084
C	-1.66226	1.19158	-0.14631
C	-1.08043	2.47817	-0.32461
C	-1.83817	3.59521	-0.50017
C	-0.9319	-1.10E-05	0.03207
C	-1.64356	-1.20975	0.15105
C	-3.05815	-1.19917	0.07879
C	-3.82928	-2.33525	0.18813
H	-4.90238	-2.22941	0.12083
C	-3.21069	-3.58491	0.38926
C	-1.78077	-3.61554	0.4947
C	-1.04297	-2.47761	0.38227
H	-4.93933	2.11996	-0.40379
H	-0.00106	2.56331	-0.3274
H	-1.34429	4.5472	-0.63585
H	-1.27429	-4.55319	0.67656
H	0.03381	-2.53033	0.4815
N	-4.01511	4.63628	-0.68583
N	-3.93626	-4.71785	0.49328
C	0.54271	0.03315	0.13036
C	1.35979	-0.62685	-0.79482
C	1.15797	0.7282	1.17436
C	2.74141	-0.55455	-0.66432
C	2.53686	0.78627	1.3487
H	0.51828	1.20037	1.91006
C	3.35878	0.14638	0.37863
H	3.36092	-0.98845	-1.43346
C	-3.28551	-6.00432	0.70697
H	-2.74753	-6.02872	1.66018
H	-4.04481	-6.78355	0.72505

H	-2.58305	-6.23328	-0.10003
C	-5.38849	-4.66096	0.41151
H	-5.71471	-4.26032	-0.55398
H	-5.79146	-5.66586	0.51878
H	-5.80613	-4.03603	1.20802
C	-3.38375	5.93943	-0.85271
H	-2.75925	5.96975	-1.75129
H	-4.15967	6.69563	-0.95265
H	-2.76697	6.19638	0.01389
C	-5.46733	4.54072	-0.7219
H	-5.88695	5.5369	-0.8455
H	-5.80379	3.91885	-1.55832
H	-5.85803	4.11619	0.20859
O	0.7326	-1.26065	-1.81885
C	1.51928	-1.95121	-2.77625
H	2.17379	-1.26473	-3.32335
H	2.12314	-2.73386	-2.3052
H	0.81289	-2.40632	-3.46976
N	3.10614	1.45739	2.4602
C	2.26943	2.42961	3.13684
H	1.46142	1.97575	3.73489
H	2.895	3.0131	3.81797
H	1.8219	3.11263	2.41076
C	3.79966	0.5871	3.40464
H	4.38705	1.19999	4.09456
H	3.09611	-0.02157	3.9951
H	4.47757	-0.07746	2.86855
N	4.75617	0.16828	0.47939
C	5.54041	-0.86799	-0.13801
H	5.02416	-1.83049	-0.06822
H	6.47877	-0.98184	0.41633
C	5.45255	1.4444	0.61406
H	6.42289	1.27238	1.09034
H	4.86831	2.10943	1.24675

H	5.61984	1.91535	-0.3622
O	-3.73135	-0.03771	-0.09141
C	5.90695	-0.6401	-1.59735
O	5.42252	0.19271	-2.32811
O	6.85135	-1.50434	-1.99329
H	7.03366	-1.33263	-2.93579

Table S5. Optimized geometry based on DFT calculation for **pHrodo** in S_1 state.

C	2.6322	-3.8889	0.5512
C	3.4224	-2.7386	0.3509
C	2.8326	-1.5016	0.1955
C	1.4365	-1.3068	0.2164
C	0.6645	-2.4678	0.4119
C	1.2304	-3.7176	0.5715
C	0.912	0.0111	0.0487
C	1.8281	1.0906	-0.1373
C	3.214	0.822	-0.1524
C	4.1684	1.801	-0.3286
H	5.2063	1.4971	-0.3168
C	3.79	3.1464	-0.5181
C	2.4086	3.4386	-0.5074
C	1.4711	2.4402	-0.3272
H	4.5022	-2.7877	0.3138
H	-0.4162	-2.3791	0.4429
H	0.5777	-4.5687	0.7124
H	2.0639	4.4552	-0.6412
H	0.4202	2.7071	-0.3293
N	3.209	-5.1239	0.7253
N	4.732	4.1269	-0.7103
C	-0.5357	0.2515	0.0937
C	-1.3701	-0.0825	-1.02
C	-1.1252	0.8496	1.1821
C	-2.7494	0.0984	-0.9588
C	-2.4968	1.1887	1.2166
H	-0.4808	1.1705	1.9878
C	-3.3577	0.638	0.1701
H	-3.3637	-0.3049	-1.7476
C	4.3219	5.5157	-0.776
H	3.8349	5.8509	0.1497
H	5.1996	6.1387	-0.9441

H	3.6276	5.6863	-1.606
C	6.14	3.8076	-0.5819
H	6.4431	3.0466	-1.3095
H	6.7285	4.7039	-0.7741
H	6.3932	3.439	0.4213
C	2.3719	-6.306	0.7836
H	1.7946	-6.4555	-0.139
H	3.0023	-7.1812	0.9373
H	1.6672	-6.2522	1.6203
C	4.639	-5.2796	0.5479
H	4.9104	-6.3184	0.7323
H	4.9652	-5.0138	-0.467
H	5.1967	-4.6585	1.2572
O	-0.7404	-0.6263	-2.0549
C	-1.4777	-0.9916	-3.2232
H	-2.1832	-1.7941	-2.9943
H	-2.0066	-0.1262	-3.6298
H	-0.7363	-1.3441	-3.937
N	-2.9611	2.0155	2.1833
C	-2.1529	2.2945	3.3642
H	-1.3601	3.0187	3.1453
H	-2.8077	2.7156	4.1271
H	-1.7135	1.3761	3.7526
C	-4.0239	2.9984	1.9684
H	-4.7991	2.8904	2.73
H	-3.584	3.9962	2.0566
H	-4.4617	2.8912	0.9811
N	-4.722	0.6645	0.2935
C	-5.5688	0.5256	-0.8691
H	-5.1302	1.0394	-1.7282
H	-6.5218	1.0228	-0.665
C	-5.3965	0.3419	1.5532
H	-6.1814	1.0735	1.7595
H	-4.6893	0.3311	2.3784

H	-5.845	-0.6522	1.473
O	3.6929	-0.4507	0.0147
C	-5.863	-0.9167	-1.2527
O	-5.3474	-1.8873	-0.7525
O	-6.7714	-0.9684	-2.2297
H	-6.9219	-1.906	-2.4527

Table S6. Optimized geometry based on DFT calculation for **pHrodoH** (*Meta*-Position) in S_0 state.

C	2.40101	4.02845	0.11544
C	3.28629	2.93884	-0.02534
C	2.79444	1.65494	-0.03172
C	1.41222	1.35177	0.0913
C	0.5387	2.46528	0.24764
C	1.00119	3.74385	0.25917
C	1.00392	0.01036	0.07317
C	1.96809	-0.99849	-0.06155
C	3.33563	-0.63831	-0.17422
C	4.34774	-1.55983	-0.30775
H	5.3615	-1.19488	-0.38681
C	4.04193	-2.93665	-0.33627
C	2.66263	-3.32226	-0.2344
C	1.68034	-2.39102	-0.10482
H	4.35325	3.07575	-0.12554
H	-0.5227	2.28363	0.36513
H	0.29744	4.55499	0.38238
H	2.39287	-4.36857	-0.26522
H	0.64781	-2.71305	-0.03956
N	2.84955	5.29782	0.12058
N	5.00941	-3.86531	-0.4603
C	-0.44077	-0.33412	0.17274
C	-0.96021	-0.88187	1.36114
C	-1.28725	-0.10972	-0.90317
C	-2.31481	-1.19602	1.44548
C	-2.634	-0.42819	-0.80671
H	-0.87667	0.30808	-1.81551
C	-3.16123	-0.97413	0.36176
H	-2.73036	-1.613	2.35374
C	4.68397	-5.28686	-0.4766
H	5.60671	-5.85767	-0.55599
H	4.17536	-5.58806	0.44406

H	4.04948	-5.54115	-1.33126
C	6.40194	-3.45475	-0.57823
H	6.7297	-2.90788	0.31196
H	7.02619	-4.33895	-0.68707
H	6.55167	-2.81804	-1.456
C	1.92688	6.4162	0.27987
H	2.49313	7.34468	0.25351
H	1.19039	6.44198	-0.52892
H	1.39996	6.36442	1.23757
C	4.27343	5.56865	-0.02567
H	4.43384	6.64451	-0.01539
H	4.84658	5.12598	0.79547
H	4.65388	5.17297	-0.97275
O	-0.07671	-1.05693	2.35954
C	-0.53014	-1.61345	3.5897
H	-1.28622	-0.97308	4.05338
H	-0.93384	-2.61892	3.43908
H	0.3482	-1.6645	4.23075
C	-3.48472	1.14323	-2.5297
H	-2.52768	1.28636	-3.02824
H	-4.29535	1.23473	-3.25094
H	-3.61162	1.86917	-1.729
C	-3.38742	-1.28722	-2.98551
H	-4.15214	-1.1363	-3.74662
H	-2.39414	-1.20531	-3.42535
H	-3.51158	-2.25992	-2.51308
N	-4.54763	-1.3035	0.39139
C	-5.41183	-0.37089	1.09655
H	-4.83739	0.36093	1.67572
H	-6.05313	-0.89082	1.81441
C	-4.84459	-2.71398	0.64761
H	-4.68029	-3.00438	1.6945
H	-4.21801	-3.33952	0.00935
H	-5.889	-2.90751	0.39331

O	3.70477	0.66318	-0.16002
C	-6.29528	0.4057	0.13974
O	-6.13329	0.45927	-1.06398
O	-7.26512	1.0535	0.77293
H	-7.77801	1.5627	0.11744
H	-4.5063	-0.32236	-1.53976
N	-3.54552	-0.22846	-1.94087

Table S7. Optimized geometry based on DFT calculation for **pHrodoH** (*Meta*-Position) in S_1 state.

C	2.1968	4.0909	0.1843
C	3.1225	3.0211	0.0617
C	2.6811	1.7256	0.019
C	1.3032	1.3696	0.0835
C	0.396	2.4575	0.2339
C	0.8104	3.7591	0.2785
C	0.9199	0.0018	0.0295
C	1.9574	-0.966	-0.0784
C	3.3167	-0.5492	-0.1233
C	4.3648	-1.4252	-0.2195
H	5.3654	-1.0174	-0.2459
C	4.1257	-2.8242	-0.288
C	2.767	-3.2663	-0.2749
C	1.7401	-2.3701	-0.1769
H	4.1878	3.195	0.0048
H	-0.6613	2.2411	0.3238
H	0.0717	4.5399	0.3965
H	2.5378	-4.3206	-0.3467
H	0.7213	-2.7366	-0.1827
N	2.6155	5.3742	0.2206
N	5.1497	-3.7003	-0.3695
C	-0.5031	-0.3968	0.0531
C	-1.0257	-1.1683	1.1141
C	-1.3646	-0.0062	-0.9673
C	-2.3655	-1.5479	1.1146
C	-2.7017	-0.3735	-0.9475
H	-0.9651	0.5863	-1.7822
C	-3.2144	-1.1594	0.081
H	-2.7662	-2.1485	1.9214
C	4.8954	-5.1323	-0.4466
H	5.8456	-5.6593	-0.492
H	4.3468	-5.4811	0.4342

H	4.3174	-5.3815	-1.3426
C	6.5269	-3.2275	-0.3849
H	6.7563	-2.661	0.5235
H	7.1957	-4.0833	-0.4362
H	6.7134	-2.5881	-1.2542
C	1.6564	6.4625	0.35
H	2.1911	7.4093	0.338
H	0.9426	6.4569	-0.4799
H	1.1016	6.3894	1.2915
C	4.0346	5.6901	0.144
H	4.1608	6.7696	0.181
H	4.5817	5.2471	0.983
H	4.4686	5.3218	-0.7913
O	-0.1542	-1.4911	2.0889
C	-0.6046	-2.287	3.1786
H	-1.4013	-1.7805	3.7318
H	-0.9559	-3.2641	2.8334
H	0.261	-2.4187	3.8258
C	-3.4729	1.4542	-2.4467
H	-2.5363	1.5746	-2.9878
H	-4.3087	1.696	-3.1015
H	-3.4944	2.0875	-1.5626
C	-3.6014	-0.9021	-3.1817
H	-4.3582	-0.5831	-3.8977
H	-2.6117	-0.8706	-3.6357
H	-3.8216	-1.9061	-2.8249
N	-4.588	-1.5671	0.021
C	-5.4341	-0.9801	1.0473
H	-4.9838	-0.9742	2.049
H	-6.3541	-1.5674	1.1253
C	-4.7482	-3.0226	-0.0817
H	-4.486	-3.5515	0.8447
H	-4.1121	-3.4008	-0.8841
H	-5.7877	-3.2472	-0.3305

O	3.653	0.773	-0.0817
C	-5.853	0.4283	0.6943
O	-5.6612	0.9679	-0.3775
O	-6.4914	1.0168	1.699
H	-6.769	1.9062	1.4094
H	-4.5626	-0.0277	-1.5636
N	-3.6276	0.0331	-2.0157

Table S8. Optimized geometry based on DFT calculation for **pHrodoH** (*para*-Position) in S_0 state.

C	2.76458	3.90868	0.05047
C	3.5663	2.75039	-0.03271
C	2.97461	1.50943	-0.04186
C	1.56873	1.31912	0.02508
C	0.7791	2.50026	0.11555
C	1.34102	3.73835	0.12717
C	1.05706	0.0146	0.01791
C	1.94109	-1.07034	-0.05834
C	3.33655	-0.82213	-0.12281
C	4.27573	-1.82406	-0.19765
H	5.31806	-1.54323	-0.24263
C	3.86099	-3.1725	-0.21172
C	2.4524	-3.44488	-0.15415
C	1.54382	-2.43599	-0.08144
H	4.64418	2.80052	-0.08741
H	-0.29819	2.40391	0.17876
H	0.69943	4.60517	0.19922
H	2.10126	-4.46695	-0.1722
H	0.48698	-2.67291	-0.04722
N	3.31223	5.1385	0.06191
N	4.75348	-4.17878	-0.28021
C	-0.41355	-0.21603	0.07455
C	-1.0063	-0.62413	1.28528
C	-1.19807	-0.0292	-1.05434
C	-2.38068	-0.84359	1.34487
C	-2.57493	-0.24799	-1.01714
H	-0.73011	0.28637	-1.98101
C	-3.12589	-0.65053	0.19314
H	-2.85399	-1.15269	2.26722
C	4.31308	-5.56914	-0.28903
H	5.18805	-6.21412	-0.33099
H	3.75198	-5.81402	0.61784

H	3.68787	-5.78391	-1.16115
C	6.17862	-3.88546	-0.34862
H	6.51187	-3.33808	0.53905
H	6.73282	-4.82006	-0.40292
H	6.41676	-3.29177	-1.23712
C	2.47535	6.33003	0.14868
H	3.11564	7.2095	0.13936
H	1.79002	6.39752	-0.70172
H	1.89289	6.34096	1.07502
C	4.75898	5.29253	-0.00973
H	5.00575	6.35171	0.01368
H	5.24994	4.80543	0.83893
H	5.15568	4.86642	-0.93688
O	-0.17008	-0.77165	2.32797
C	-0.70137	-1.19008	3.58119
H	-1.427	-0.46417	3.95997
H	-1.16679	-2.17687	3.49899
H	0.14891	-1.24314	4.25874
C	-3.55601	1.29219	-2.60712
H	-2.62541	1.63412	-3.08213
H	-4.36222	1.35467	-3.34153
H	-3.79031	1.95839	-1.77474
C	-3.19159	-1.02672	-3.22548
H	-3.99982	-0.95197	-3.95646
H	-2.24144	-0.81135	-3.73462
H	-3.16101	-2.04893	-2.84367
N	-4.57711	-0.86371	0.17247
C	-5.33511	0.08099	1.04399
H	-4.80791	1.03622	1.07481
H	-5.39659	-0.31657	2.05743
C	-4.97083	-2.29016	0.40828
H	-4.72282	-2.56251	1.433
H	-4.42358	-2.91855	-0.29147
H	-6.04171	-2.37814	0.23516

O	3.80728	0.44619	-0.11526
C	-6.7118	0.28873	0.44827
O	-7.02189	-0.1122	-0.64857
O	-7.49013	0.97492	1.27006
H	-8.35483	1.11622	0.83929
N	-3.46241	-0.09109	-2.12884
H	-4.81049	-0.62399	-0.82514

Table S9. Optimized geometry based on DFT calculation for **pHrodoH** (*para*-Position) in S_1 state.

C	2.7001	3.9542	0.2238
C	3.5186	2.8002	0.1012
C	2.9538	1.5541	0.046
C	1.5476	1.3329	0.0991
C	0.7485	2.5029	0.2462
C	1.2873	3.7578	0.3027
C	1.035	0.0084	0.0381
C	1.9742	-1.0541	-0.0742
C	3.3678	-0.7701	-0.1139
C	4.3265	-1.7421	-0.2211
H	5.3618	-1.4319	-0.244
C	3.9534	-3.1103	-0.3079
C	2.5583	-3.4191	-0.2955
C	1.6227	-2.4295	-0.1856
H	4.5964	2.8687	0.0529
H	-0.3258	2.3896	0.3219
H	0.6269	4.6063	0.4175
H	2.2279	-4.4453	-0.3778
H	0.5733	-2.6966	-0.1936
N	3.2413	5.1905	0.2724
N	4.8866	-4.0811	-0.406
C	-0.4208	-0.2502	0.0529
C	-1.0136	-0.9664	1.1182
C	-1.2332	0.2158	-0.9766
C	-2.3845	-1.212	1.1255
C	-2.608	-0.011	-0.9869
H	-0.7778	0.7591	-1.7984
C	-3.1464	-0.7262	0.0749
H	-2.8465	-1.7592	1.9365
C	4.492	-5.4791	-0.5114
H	5.385	-6.0948	-0.5891
H	3.9272	-5.7955	0.3716

H	3.8768	-5.6474	-1.4012
C	6.3035	-3.7459	-0.4144
H	6.5837	-3.2092	0.4977
H	6.886	-4.6627	-0.467
H	6.5551	-3.1233	-1.2797
C	2.3925	6.3673	0.3983
H	3.0193	7.256	0.4082
H	1.6976	6.4424	-0.4444
H	1.8154	6.3395	1.3285
C	4.6847	5.3669	0.205
H	4.9158	6.4284	0.2551
H	5.1816	4.8634	1.0411
H	5.0858	4.9684	-0.7327
O	-0.1775	-1.3712	2.0937
C	-0.7023	-2.1239	3.1804
H	-1.4426	-1.5432	3.7395
H	-1.1501	-3.0596	2.8314
H	0.1472	-2.3451	3.8245
C	-3.6133	1.8859	-2.1049
H	-2.6881	2.3502	-2.4752
H	-4.4248	2.136	-2.7923
H	-3.8458	2.3037	-1.1236
C	-3.2294	-0.1759	-3.3219
H	-4.0453	0.0758	-4.0033
H	-2.2881	0.1879	-3.758
H	-3.1747	-1.2621	-3.2286
N	-4.596	-0.9417	0.0093
C	-5.3549	-0.2645	1.1009
H	-4.8321	0.6523	1.3786
H	-5.4093	-0.9124	1.9761
C	-4.9753	-2.3839	-0.1327
H	-4.697	-2.9145	0.7765
H	-4.4422	-2.7965	-0.9867
H	-6.0497	-2.4383	-0.2976

O	3.8297	0.5127	-0.0558
C	-6.7356	0.0837	0.5862
O	-7.0538	-0.0269	-0.5743
O	-7.5089	0.5383	1.56
H	-8.3761	0.7833	1.1843
N	-3.5061	0.4263	-2.0136
H	-4.8391	-0.4515	-0.8892

References

(1) Gaussian 09, Revision E.01, Frisch, M. J. G. W. Trucks, H. B. Schlegel, G. E. Scuseria, M. A. Robb, J. R. Cheeseman, G. Scalmani, V. Barone, B. Mennucci, G. A. Petersson, H. Nakatsuji, M. Caricato, X. Li, H. P. Hratchian, A. F. Izmaylov, J. Bloino, G. Zheng, J. L. Sonnenberg, M. Hada, M. Ehara, K. Toyota, R. Fukuda, J. Hasegawa, M. Ishida, T. Nakajima, Y. Honda, O. Kitao, H. Nakai, T. Vreven, J. A. Montgomery, Jr., J. E. Peralta, F. Ogliaro, M. Bearpark, J. J. Heyd, E. Brothers, K. N. Kudin, V. N. Staroverov, T. Keith, R. Kobayashi, J. Normand, K. Raghavachari, A. Rendell, J. C. Burant, S. S. Iyengar, J. Tomasi, M. Cossi, N. Rega, J. M. Millam, M. Klene, J. E. Knox, J. B. Cross, V. Bakken, C. Adamo, J. Jaramillo, R. Gomperts, R. E. Stratmann, O. Yazyev, A. J. Austin, R. Cammi, C. Pomelli, J. W. Ochterski, R. L. Martin, K. Morokuma, V. G. Zakrzewski, G. A. Voth, P. Salvador, J. J. Dannenberg, S. Dapprich, A. D. Daniels, O. Farkas, J. B. Foresman, J. V. Ortiz, J. Cioslowski, and D. J. Fox, Gaussian, Inc., Wallingford CT, 2013.

(2) Yanai, T.; Tew, D. P.; Handy, N. C. A new hybrid exchange–correlation functional using the Coulomb-attenuating method (CAM-B3LYP). *Chem. Phys. Lett.* **2004**, *393*, 51-57.

(3) Rassolov, V. A.; Pople, J. A.; Ratner, M. A.; Windus, T. L. 6-31G* basis set for atoms K through Zn. *J. Chem. Phys.* **1998**, *109*, 1223-1229.

(4) Cammi, R.; Tomasi, J. Remarks on the use of the apparent surface charges (ASC) methods in solvation problems: Iterative versus matrix-inversion procedures and the renormalization of the apparent charges. *J. Comput. Chem.* **1995**, *16*, 1449-1458.

(5) S. Miertuš; E. Scrocco; Tomasi, J. Electrostatic interaction of a solute with a continuum. A direct utilizaion of AB initio molecular potentials for the prevision of solvent effects. *Chem. Phys.* **1981**, *55*, 117-129.

(6) K. Lawson-Wood, S. Upstone, K. Evans, *Fluoresc. Spectrosc.***2018**, *4*, 1-5.

(7) Snellenburg, J. J.; Laptenok, S.; Seger, R.; Mullen, K. M.; van Stokkum, I. H. M., Glotaran: A Java-Based Graphical User Interface for the R Package TIMP. *J. Stat. Softw.* **2012**, *49*, 1 - 22.
