# Supplementary material for: Sensing Mechanism and Excited-State Dynamics of a Widely Used Intracellular Fluorescent pH Probe: pHrodo
Source: J Phys Chem Lett. 2023 Nov 15;14(46):10482–8. doi: 10.1021/acs.jpclett.3c02653 (PMC10683063; doi:10.1021/acs.jpclett.3c02653)
Supplement: Supplementary file 2 — jz3c02653_si_002.pdf [file jz3c02653_si_002.pdf]

jz-2023-02653m.R1

Name: Peer Review Information for "Sensing Mechanism and Excited-State Dynamics of a Widely Used Intracellular Fluorescent pH Probe: pHrodo"

First Round of Reviewer Comments

Reviewer: 1

Comments to the Author

The authors have studied the sensing mechanism of a widely used fluorescent pH probe (pHrodo) with a focus on its excited-state dynamics by means of ultra-fast optical spectroscopy and theoretical calculations. There are numerous efforts to develop new fluorescent probes based on a “trial and error” approach. However, there is little emphasis on their sensing mechanisms, especially their photophysics. A comprehensive understanding of their sensing mechanisms is beneficial for the rational design of new fluorescent probes. Hence, the current work is very important for the field by offering significant scientific advances and physical insight into the widely used pH probe (pHrodo). The results presented in the work will make far-reaching impacts in the general field of fluorescent molecular materials. Furthermore, the work is well done, and the manuscript is well written and needs just minor revision (see my comments below) to be published in the Journal of Physical Chemistry Letters.

1. In Figure 2, the authors calculated the RMSD values of pHrodo and pHrodoH. But a clear description/definition of RMSD is missing in the manuscript.

2. As intramolecular charge transfer process is involved, it would be much better if the authors could give the dipole moments of ground state and first excited state.

3. In Figure 5, the authors can consider adjusting the time-axis in (c), so that the readers can see the fast decay component (1ps).

4. The authors measured the fluorescence quantum yield by using Rhodamine 6G (PLQY=0.95) as the reference. They need to provide a reference for the fluorescence quantum yield of Rhodamine 6G. By the way, PLQY is not defined in the supplementary information or manuscript.

Reviewer: 2

Comments to the Author

The manuscript reports on the excited state dynamics of a commercial fluorescent pH probe used to map intracellular pH. Supported by steady state and time-resolved spectroscopy and TDDFT calculations, the authors elucidate the pH sensing mechanism of pHrodo.

The proposed bichromophoric model used to understand the pH sensing mechanism is supported by the observation of a weak and broad absorption band due to a weakly allowed CT state. Together with an earlier report on aminorhodamine, evidence accumulates to show that such mechanism could be extended to other fluorescent probes.

However, in the present form the discussion and conclusions extracted from the fs-TA data of the neutral form of pHrodo does not appear to be supported by the data. In particular, on page 9 the discussion about the initial ESA absorption at 480 nm that decays and the buildup of the ESA band at 440 nm is hard to follow, because such dynamics is not at all evident in Fig 3a, b or c. I suggest zooming in the time scale to shown only the first 2 ps in panel a and c. The data should show unambiguously the buildup of the ESA of S1 state, otherwise the data does not appear to support the discussion.

In addition:

1) The manuscript should highlight in the introduction what are the differences between the proposed model and the TICT model to clarify the assumptions upon which the bichromophoric models is based on.

2) The manuscript should clarify how the RMSD was calculated

3) On page 7, FOM should be FMO

Reviewer: 3

Comments to the Author

In the work by Chen et. al., femtosecond transient absorption spectroscopy and quantum chemistry calculations were used to study the excited-state dynamics of a widely used fluorescent intracellular pH probe: pHrodo. They addressed the sensing mechanism of the widely used pH probe, which offers fundamental physical understanding and thus will make an extensive influence in the field of organic fluorescent molecules and probes, especially for new fluorescent probes' development. In my opinion, the work is well done and presented. The manuscript should be accepted for publication with some minor revisions.

1. The molecular structures in Figure 1 (a) and (c) should not be filled with orange color. The current format will make readers confused.

2. In Figure 2, the authors should label the energy (in eV) of each FMO. The RMSD is not clearly defined in the manuscript. S0 and S1 should be labeled at the lower panel of Figure 2 and Figure S5.

3. I suggest that the authors try to discuss why the decay process from S1 to S0 of pHrodo (0.5 ps) is much faster than that of TMARh (1.7 ps reported in Phys. Chem. Chem. Phys., 2022, 24, 26731-26737).

4. Give the fitting function in Figure S1 (c) and (f).

5. The authors should use the same terminology for the manuscript and SI, such as “fluorescence”, “photoluminescence”, “FQY” and “PLQY”.

6. The dihedral angle should be defined with the four atoms involved in Figures S2-S4.

Author's Response to Peer Review Comments:

Thank you very much for all the help with our manuscript entitled "**Sensing Mechanism and Excited-State Dynamics of a Widely Used Intracellular Fluorescent pH Probe: pHrodo**" (Manuscript ID: jz-2023-02653m). We also thank the referees for their careful reading of the manuscript, constructive comments, and suggestions. We have studied their comments carefully and revised the manuscript accordingly. All changes are highlighted in red color in the revised manuscript. Please find the attached point-by-point responses to the comments.

Best regards,

Junsheng Chen, on behalf of co-authors

Nano-Science Center & Department of Chemistry, University of Copenhagen, Universitetsparken 5, DK-2100 Copenhagen, Denmark

EDITORIAL COMMENTS:

(1) You should find a way to reduce the number of figures in the main text to be closer to the standard letter size of 4 figures. You may move a figure to the SI or combine figures if that is easier, but 6 is too many.

Response: We have moved the previous Figure 2 and Figure 4 to the Supporting Information as Figure S2 and Figure S9. Now there are 4 figures in the revised manuscript.

(2) Supporting Information Statement: A brief, non-sentence description of the actual contents of each supporting information file is required. This description should be labeled Supporting Information and should appear before the Acknowledgement and Reference sections.

Response: We have added a brief description of each supporting information before the Acknowledgement section.

(3) Headers: Remove the section heading(s) throughout the body of the manuscript (you can leave Methods, Abstract, and TOC Graphic headings).

Response: Amended.

(4) Title and Author Lists: Title, author names, and affiliations must match in three places: (1) manuscript file, (2) supporting information, and (3) ACS Paragon Plus.
Response: Amended.

(5) References: In both the main file and the supporting information, fix the style of all references to use JPCL formatting (check all references carefully). ***JPC Letters reference formatting requires that journal references should contain: () around numbers; author names; article title (titles entirely in title case or entirely in lower case); abbreviated journal title (italicized); year (bolded); volume (italicized); and pages (first-last). Book references should contain author names; book title (in the same pattern); publisher; city; and year. Websites must include date of access.

Response: Amended.

(6) References: URLs are not preferred references because website content can be modified and, consequently, the reference information may lack permanence. **Response:** We revised the format of reference 29 with more detailed information.

(7) Graphics: One or more of your figures and tables includes a reference citation. Please confirm that this pertains only to data and not the figure itself. If it pertains to the use of a published image, permissions must be secured for any graphics NOT originally published by ACS or for Open Access content which permits reuse with credit only. Permission is needed if you are using another publisher's or copyright owner's figures/tables verbatim, adapting/modifying them, or using them in part. If the images are from an Open Access publisher that does not require permission for reuse, please confirm.

Response: We included one reference citation about the molecular structure of pHrodo in Figure 1. We confirm this pertains only to the data and not the figure itself. We moved the relevant description and reference from the figure caption to the main text (page 4 and page 6 of the revised manuscript).

Point-by-point responses to reviewers' comments

Reviewer 1

The authors have studied the sensing mechanism of a widely used fluorescent pH probe (pHrodo) with a focus on its excited-state dynamics by means of ultra-fast optical spectroscopy and theoretical calculations. There are numerous efforts to develop new fluorescent probes based on a "trial and error" approach. However, there is little emphasis on their sensing mechanisms, especially their photophysics. A comprehensive understanding of their sensing mechanisms is beneficial for the rational design of new fluorescent probes. Hence, the current work is very important for the field by offering significant scientific advances and physical insight into the widely used pH probe (pHrodo). The results presented in the work will make far-reaching impacts in the general field of fluorescent molecular materials. Furthermore, the work is well done, and the manuscript is well written and needs just minor revision (see my comments below) to be published in the Journal of Physical Chemistry Letters.

We thank the reviewer for the positive reception of our work and acknowledge the importance of studying the sensing mechanisms of fluorescent sensors.

1. In Figure 2, the authors calculated the RMSD values of pHrodo and pHrodoH. But a clear description/definition of RMSD is missing in the manuscript.

Response: The root-mean-squared deviation (RMSD) is a value that can be used to represent the change between two structures. Here, we used RMSD value to quantitatively describe the total molecular structural change between ground state and the first excited state, which includes changes of bond length, bond angle and dihedral angle. The description of RMSD is added in our revised manuscript (page 7) and SI (page S2).

2. As intramolecular charge transfer process is involved, it would be much better if the authors could give the dipole moments of ground state and first excited state.

Response: The calculated dipole moments of the ground state and first excited state are added in Figure S7. The dipole moment of pHrodo is decreased from the ground state (8.68 D) to the first excited state (6.87 D). The decrease of dipole moment is because of pHrodo is cationic with charge (positive) localization on the diamion-xanthium part. In S_1, the charge (negative) transfer from the arylpyrylium chromophore part to the cationic diamion-xanthium part, as a result, the dipole

moment of pHrodo is decreased to 6.87 D. This confirms the charge separation nature of the first excited state. We added relevant discussion in the revised manuscript, please see it on page 7.

3. In Figure 5, the authors can consider adjusting the time-axis in (c), so that the readers can see the fast decay component (1ps).

Response: We replotted the Figure 5c in manuscript (Figure 3c in the revised manuscript) to make the fast decay component clearer.

4. The authors measured the fluorescence quantum yield by using Rhodamine 6G (PLQY=0.95) as the reference. They need to provide a reference for the fluorescence quantum yield of Rhodamine 6G. By the way, PLQY is not defined in the supplementary information or manuscript.

Response: We have cited the related reference for the fluorescence quantum yield of Rhodamine 6G (reference 6 in SI). We replaced PLQY with FQY throughout the manuscript and SI.

Reviewer 2

The manuscript reports on the excited state dynamics of a commercial fluorescent pH probe used to map intracellular pH. Supported by steady state and time-resolved spectroscopy and TDDFT calculations, the authors elucidate the pH sensing mechanism of pHrodo.

The proposed bichromophoric model used to understand the pH sensing mechanism is supported by the observation of a weak and broad absorption band due to a weakly allowed CT state. Together with an earlier report on aminorhodamine, evidence accumulates to show that such mechanism could be extended to other fluorescent probes.

However, in the present form the discussion and conclusions extracted from the fs-TA data of the neutral form of pHrodo does not appear to be supported by the data. In particular, on page 9 the discussion about the initial ESA absorption at 480 nm that decays and the buildup of the ESA band at 440 nm is hard to follow, because such dynamics is not at all evident in Fig 3a, b or c. I suggest zooming in the time scale to shown only the first 2 ps in panel a and c. The data should show unambiguously the buildup of the ESA of S1 state, otherwise the data does not appear to support the discussion.

Response: As suggested by the referee, we zoomed in the time scale to show the first 2 ps (Figure S7), in which we can observe the ESA signal at 480 nm builds up within an ultrafast timescale, and followed with a fast decay. While the trace of ESA signal at 440 nm gradually builds up in 100 fs. The internal conversion process from S_2 to S_1 happens in an ultrafast time scale (<100 fs), which is faster than the instrument response function (IRF) of our fs-TA. As a result, the buildup process of the ESA band at 440 nm cannot be fully resolved here. But the internal conversion process is supported by the decay process at 480 nm being faster than that at 440 nm (Figure S7). Furthermore, such ultrafast process is confirmed by the global analysis: the <100 fs component (Figure 2d).

We would like to emphasis that the bichromophoric model and the existence of the S_1 state are further confirmed by 640 nm excitation fs-TA measurement (Figure S8), a weak broad absorption band in the steady-state absorption spectrum (Figure 1d), the presence of the fluorescence at low temperature, and theoretical calculations. We added description of the zoomed in plot in the revised manuscript (page 9).

In addition:

1) The manuscript should highlight in the introduction what are the differences between the proposed model and the TICT model to clarify the assumptions upon which the bichromophoric models is based on.

Response: We thank the referee for the good suggestion. We added a description about the difference between bichromophore model and TICT model in the introduction, please see on page 4 in the revised manuscript.

2) The manuscript should clarify how the RMSD was calculated

Response: Please see the response to the second comment from reviewer 1.

3) On page 7, FOM should be FMO

Response: Amended.

Reviewer 3

In the work by Chen et. al., femtosecond transient absorption spectroscopy and quantum chemistry calculations were used to study the excited-state dynamics of a widely used fluorescent intracellular pH probe: pHrodo. They addressed the sensing mechanism of the widely used pH probe, which offers fundamental physical understanding and thus will make an extensive influence in the field of organic fluorescent molecules and probes, especially for new fluorescent probes' development. In my opinion, the work is well done and presented. The manuscript should be accepted for publication with some minor revisions.

We thank the reviewer for the positive feedback on our manuscript and for acknowledging important aspects of our study.

1. The molecular structures in Figure 1 (a) and (c) should not be filled with orange color. The current format will make readers confused.

Response: Amended.

2. In Figure 2, the authors should label the energy (in eV) of each FMO. The RMSD is not clearly defined in the manuscript. S0 and S1 should be labeled at the lower panel of Figure 2 and Figure S5.

Response: Please see the response to the second comment from reviewer 1. We labeled the energy of each FMO, color code for S0 and S1 in Figures S2 and S6 (previously Figure 2 and Figure S5).

3. I suggest that the authors try to discuss why the decay process from S1 to S0 of pHrodo (0.5 ps) is much faster than that of TMARh (1.7 ps reported in Phys. Chem. Chem. Phys., 2022, 24, 26731-26737).

Response: We thank the reviewer for this observation. The fast decay process from S_1 to S_0 involves structural relaxation, especially the dihedral angle change between the two planes of arylpyrylium and diamion-xanthium. As shown in Figure R1 on next page: In TMARh, the presence of four methoxy groups hinders such structural relaxation; In pHrodo, there is only one methoxy group. Hence, the structural relaxation of pHrodo is less restricted compared to that of TMARh. This is why the decay process from S_1 to S_0 of pHrodo is much faster than that of TMARh.

We added relevant discussion in the revised manuscript. Please see it on page 11.

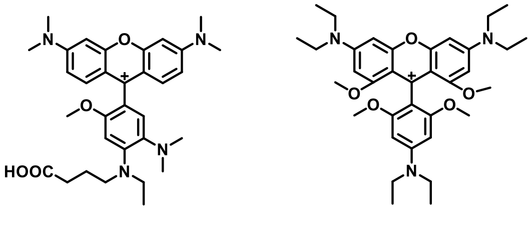

Figure R1. Molecular structures of pHrodo and TMARh.

4. Give the fitting function in Figure S1 (c) and (f).

Response: We added the fitting function in Figure S1c and S1f.

5. The authors should use the same terminology for the manuscript and SI, such as "fluorescence", "photoluminescence", "FQY" and "PLQY".

Response: We have replaced the "photoluminescence" with "fluorescence", and "PLQY" with "FQY" in the manuscript and SI.

6. The dihedral angle should be defined with the four atoms involved in Figures S2-S4.

Response: The dihedral angle is defined by the planes between arylpyrylium and diamion-xanthium. In our analysis, we drew two planes, which cross arylpyrylium and diamion-xanthium, and we measured the angle between the two planes, instead of measuring the dihedral angle by selecting four atoms. We added more description in SI.
